# When There's No November Rain: Developing a Parametric Insurance for Hydroelectric Energy Generators in Brazil

**DOI:** 10.1111/risa.70347

**Published:** 2026-09-03

**Authors:** Nathalia Costa Fonseca, João Vinícius França Carvalho, Thiago Dutra Araújo, André Luis Squarize Chagas

**Affiliations:** ^1^ Department of Accounting and Actuarial Science; ^2^ Department of Economics University of São Paulo – FEA/USP São Paulo Brazil

**Keywords:** copulas, energy generators, hydrological risk, parametric insurance, spatial econometrics

## Abstract

Hydrological risk is a growing challenge for renewable power systems, as droughts reduce generation, increase costs, and expose gaps in risk‐transfer mechanisms. We examine this problem in Brazil, home to the world's sixth‐largest electricity system and highly dependent on hydroelectricity, which accounted for 43% of installed generation capacity in 2026. We develop and evaluate a parametric insurance scheme for run‐of‐river hydroelectric generators indexed to the National System Operator's affluent flow measure. Using monthly energy and climate data from 151 powerplants between 2006 and 2022, we incorporate hydrological network dependence into insurance design. We model generation through a time‐varying upstream–downstream spatial network and estimate it using a two‐way fixed‐effects SAR‐IV‐GMM‐HAC specification. We then use vine copulas to assess the dependence structure among climatic variables, hydrological conditions, and electricity generation, supporting the use of affluent flow as the insurance trigger. Results reveal significant hydrological propagation across connected plants and indicate that parametric insurance can mitigate drought‐related losses. However, feasibility depends on plant‐specific trigger design, diversification, reinsurance support, and payout magnitude. Water‐storage plants exhibit higher basis risk because of their operational flexibility.

## 1 Introduction

The nonlife insurance industry is crucial for safeguarding society against a myriad of perils. However, not all risks are insurable (for their nature or the insurers’ underwriting choices) and not all insurable risks are insured (Lin and Kwon 2020; Schanz 2018). This is reflected by a concept called *insurance protection gap* (IPG), meaning “the difference between the amount of insurance that is economically beneficial and the amount of coverage actually purchased” (Schanz 2018, 1), which is common for fundamental risks (Singer 2019).

Parametric insurance (PI), also known as index‐based insurance, offers a promising solution to IPG problems while improving market efficiency (Lin and Kwon 2020). Unlike indemnity‐based insurance, PI does not cover the pure loss, but rather indemnifies the policyholder according to an index (parameter) variation, which is observable, reliable, and ideally correlated with the sustained losses (Singer 2019). The PI payouts are only triggered by the index, and the payout value is agreed upon in advance, instead of based on the actual losses incurred (Enríquez et al. 2020). PI is particularly useful when information is insufficient to underwrite the severity of actual losses, also overcoming the problem of information asymmetry, reducing adverse selection and preventing moral hazards, while not requiring claim investigations (Bokusheva 2018; Han et al. 2019).

PI enables coverage for risks that are difficult to insure through traditional indemnity contracts (Lin and Kwon 2020). PI solutions usually do not protect against direct physical damage, but rather the indirect consequences of events, such as business interruption costs (Singer 2019). One application of PI relates to weather issues, especially under climate change (Horton 2018).

Climate change is a current world concern as it has been causing multiple changes in various regions, which shall increase with further warming. Such extreme events have the potential to cause billionaire damages, concerning governments and institutions worldwide (Mendes‐Da‐Silva et al. 2021). Thus, numerous applications of PI have arisen addressing climate change problems. Insurance for agriculture is a common application (Abdi et al. 2022; Horton 2018; Wijesena and Pradhan 2026), but there is also a growing use of parametric schemes for natural disasters (Bai and Chen 2026; Men et al. 2025; Steinmann et al. 2023) and an innovation is regarding renewable energy generation.

Examples of the latter include (i) wind farms, as the energy generation is affected by absent or low‐speed wind, conditions affected by climate change; (ii) hydroelectric powerplants, as reservoirs are susceptible to droughts that reduce their water storage, and even; (iii) photovoltaic energy is susceptible to weather conditions, as the electricity generation depends on the solar radiation flux that reaches the cells, which can be affected by cloudy, rainy, or dusty conditions during daylight hours (Boyle et al. 2021; Han et al. 2019; Scarpellini et al. 2025). Demand for renewable‐energy PI should grow with renewable expansion and climate‐risks concerns (Enríquez et al. 2020).

In this study, we aim to evaluate the costs and viability of a PI scheme for renewable energy generation in Brazil. Bestowed with the world's largest fresh water reserves, Brazil's energy consumption predominantly relies on hydroelectric power. However, the nation has grappled with adverse impacts of climate change, notably prolonged periods of severe drought (Hochberg and Poudineh 2021).

Droughts are not a new problem in Brazil, however, climate change has increased their intensity and frequency (Paim et al. 2019). There is a high cost in dealing with energy crises resulting from drought, as thermoelectric powerplants need to be actioned (and besides being costlier, many of them run on fossil fuels) and sometimes even electricity rationing is imposed. These costs hit society by elevating the electricity tariffs and can worsen economic crises already present in the nation (Hunt et al. 2018).

Our study proposes a PI product design for Brazilian run‐of‐river hydroelectric generators, indexed on the National System Operator (ONS)’s affluent flow index, which could improve the companies’ operational sustainability and mitigate effects of drought crises. The product follow established PI designs regarding both contractual structure and actuarially fair pricing. Our contribution is to incorporate hydrological network dependence into PI design for hydropower generation.

Although PI applications in renewable energy are growing, applications involving hydropower generation remain relatively limited, partly because large‐scale hydropower generation is concentrated in a few major producing countries and depends on favorable geographic, hydrological, and climatic conditions (IEA). The Brazilian case provides an especially relevant setting, as the country holds the sixth largest electricity system globally^1^ and is highly dependent on hydroelectricity. As of 2026, Brazil's generation installed capacity is distributed as follows: 43% hydro, 19% small‐scale distributed generation, 14% wind, 9% thermal (coal, oil, and natural gas), 8% solar, 6% biomass, and 1% nuclear.^2^

We model hydropower interdependence through a time‐varying hydrological network based on first‐order upstream–downstream links. This framework captures dependence among plants within the same river system and shows that electricity generation depends on local conditions and hydrological interactions.

As a robustness check, we use vine copulas to characterize dependence (Czado 2025) among climatic variables, hydrological flow, and electricity generation. Vine‐copula results indicate that affluent flow is the most suitable trigger for run‐of‐river plants because it is statistically associated with electricity generation through the hydrological process. This pattern is absent in water‐storage plants, whose operational flexibility weakens the link between the trigger index and insured losses. Accordingly, the proposed PI scheme is restricted to run‐of‐river plants.

## 2 Theoretical Background

Brazil hosts one of the most extensive electricity systems globally,^3^ with three of the world's longest power transmission lines.^4^ The country has one of the most renewable energy matrices in the world (Corrêa and Maya 2023). Brazil's geographic and climatic conditions, with abundant water resources, historically favored investments in hydroelectricity (Cataia 2019), which remains its main source of energy generation, despite the country's great potential for exploring other renewable sources, such as wind, solar, and biomass (Corrêa and Maya 2023).

Hydroelectric plants are generally classified as run‐of‐river and water storage. Run‐of‐river powerplants utilize the natural flow of a river, while water‐storage plants store water in natural or artificial reservoirs and release it through the turbines according to the electricity demand.^5^ Water‐storage powerplants can release or store water according to demand, providing greater operational flexibility.

Hydroelectricity stands as a vital renewable technology, making a significant contribution to grid regulation, as it quickly and effectively responds to fluctuations in electricity demand.^6^ However, this type of energy source is subjected to climate fluctuations, and it is especially vulnerable to drought periods (Hunt et al. 2018).

Diversification of the electricity matrix is an approach that can contribute to reduce hydrological and economic risks and has been stimulated over the last few decades in Brazil (Paim et al. 2019). Furthermore, Brazil has designed its own regulatory mechanism: the Energy Reallocation Mechanism (MRE). This framework is designed to apportion hydrological risks among energy generators, facilitating more effective management of water reservoir levels and mitigating the financial vulnerability of energy producers (Paim et al. 2019).

MRE serves as an accounting mechanism, intended to promote the financial sharing of hydrological risk by creating an accounting portfolio of hydroelectric production, which leverages the diversity of hydrological patterns in Brazilian watersheds. However, it does not function as a tool capable of mitigating systemic risk; its primary role is to reduce the volatility to which an individual hydroelectric generator is exposed (Paim et al. 2019). During periods of water scarcity, when hydroelectric powerplants cannot meet the energy demand, other sources, typically thermoelectric, are required to compensate. This prompts a significant economic cost for both the generators, obliged to purchase the shortfalls at market price, and the consumers, as energy tariffs rise (Hunt et al. 2018).

Insurance can mitigate the financial impacts of climate change (Horton 2018). Recent literature documents numerous PI applications addressing climate risks. Insurance for agriculture is the most common application, as the first parametric schemes were focused on this theme (Horton 2018). Abdi et al. (2022) reviewed the available literature on weather index insurance for agricultural proposes, between 2001 and 2020, and found that rainfall and temperature‐based indices were most prevalent, while indices based on droughts, floods, vegetation, soil moisture, humidity, and sunshine hours were underrepresented despite their demonstrated potentials.

Kusuma et al. (2018) developed a drought‐index insurance scheme for rice production in Indonesia using geographically weighted regressions and actuarial pricing, demonstrating the feasibility of location‐specific insurance design. It shows effectiveness in some districts, but not in all.

Another applicability of index‐based insurance is regarding natural disasters and associated social matters. Figueiredo et al. (2018), for instance, proposed a generic probabilistic framework for parametric trigger modeling based on logistic regression and applied it to flood‐related disasters in Jamaica, from 1998 to 2016, using precipitation data as index. The model has substantial skill at predicting the probability of loss occurrence on each day. They also demonstrated that the system can provide considerable utility to involved parties.

Regarding energy generation applications, Han et al. (2019) studied the weather risks faced by wind‐power producers in China, and discussed the feasibility of a wind‐power generation PI scheme. Their simulation results show that wind‐power enterprises can effectively avoid economic losses caused by weather risks through PI. More recently, Scarpellini et al. (2025) developed a framework to design and evaluate PI schemes for the hydroelectric sector, comparing alternative indices and contract structures in the Lake Como Basin. Their results emphasize the importance of index design for reducing basis risk and improving insurance performance.

Moreover, Enríquez et al. (2020) provided a report, focused on Central America, describing the characteristics, (dis)advantages, and potential markets for PI for renewable energy producers. Unlike some developing regions discussed by Enríquez et al. (2020), Brazil combines a large renewable‐energy market (Montoya et al. 2021), reliable meteorological data from the National Meteorology Institute (INMET) (de Aguiar and Lobo 2020), and a mature insurance sector (de França Carvalho and Guimarães 2024), making it a particularly suitable environment for PI applications.

Most studies combine historical weather indices and economic outcomes to construct actuarial pricing models. Most empirical studies have relied on OLS‐based correlations or linear regression techniques to determine insurance premiums (Abdi et al. 2022).

Recent studies have explored more flexible methodologies for PI design. Cesarini et al. (2021) and Men et al. (2025) show the usefulness of machine‐learning approaches for identifying extreme events and predicting insurance losses, while Bokusheva (2018) highlights the advantages of copulas for modeling tail dependence and reducing basis risk.

Motivated by Brazil's hydropower dependence, we develop a PI scheme for Brazilian run‐of‐river hydroelectric powerplants using affluent flow as the trigger. Pricing combines spatial econometrics to account for the location‐dependent relationship between hydrometeorological variables and electricity generation, complemented by a vine‐copula analysis of the variables’ dependence structure. This approach allows portfolio risk to be evaluated through the hydrological network connecting powerplants rather than through generic geographic proximity.

## 3 Methodology

The calculation of the risk premium, based on Han et al. (2019) and Kusuma et al. (2018), is as follows. First, a probability distribution is fitted on historical empirical data. Then, the model parameters are estimated. Finally, the expected payout value is weighted by the probabilities of occurrence, resulting in the risk premium ($P_{m}$):

(1) $$P_{m}=\frac{1}{M}\sum_{m=1}^{M}\left(S\left(I_{w,m}\right)\times P\left(I_{w,m}\right)\right),$$

where *M* is the total number of months during the insurance term, $I_{w,m}$ represents the generation capacity of the powerplant at the *m*th month, and $S(I_{w,m})$ represents the payout value at the *m*th month. This equation provides monthly pricing and can be extended to the contract term by multiplying $P_{m}$ by *M*. Because PI is typically short‐term, risk‐free discounting can be neglected.

The payout value, $S(I_{w,m})$, combines historical observed losses and loss occurrence probability, as is standard in insurance pricing (Frees 2015). In the proposed PI scheme, frequency is estimated by fitting a probability distribution to the chosen parameter. Severity is derived from historical generation levels and on‐grid energy price, which is why the payout is expressed in terms of the powerplants’ generation capacity ($I_{w,m}$).

In that way, following Kusuma et al. (2018), we define the payout as:

(2) $$S\;\left(I_{w,m}\right)=G_{w}\;\times \;\left(I_{w,m}\right)=G_{w}\;\times \left\{\begin{array}{cc} 0 & A_{o,m}>A_{Tr}\\ \beta \left(A_{Tr}-A_{o,m}\right) & A_{E}<A_{o,m}<A_{Tr}\\ \beta (A_{E}-A_{o,m}) & A_{o,m}<A_{E}<A_{Tr,} \end{array}\;\right.\;$$

where $G_{w}$ is the on‐grid power price, $A_{o,m}$ is the observed affluent flow at each month *m*, $A_{Tr}$ is the affluent flow trigger, and $A_{E}$ is the affluent flow exit threshold. β is a parameter representing the direct effect coefficient estimated for *A* in the econometric model.

Thus, payouts increase as affluent flow falls below the trigger until reaching the exit threshold, beyond which it remains constant, serving as a maximum retention limit value.

Given the trigger and exit thresholds, $P(I_{i})$ is

(3) $$P\;\left(I_{i}\right)=F\left(A_{Tr}\right)-F\left(A_{E}\right),$$

where $F\;\left(A_{Tr}\right)=P\left(A\leq A_{Tr}\right)$ is the cumulative distribution function, which measures the probability of the affluent flow level *A* being less than $A_{Tr}$. Hence, the insurance monthly premium is

(4) $$P_{m}=\frac{1}{M}\;\sum_{m=1}^{M}G_{w}\times \left(F\left(A_{Tr}\right)-F\left(A_{E}\right)\right)\times \left\{\begin{array}{cc} 0 & A_{o,m}>A_{Tr}\\ \beta \left(A_{Tr}-A_{o,m}\right) & A_{E}<A_{o,m}<A_{Tr}\\ \beta (A_{E}-A_{o,m}) & A_{o,m}<A_{E}<A_{Tr}. \end{array}\right.$$

Following Kusuma et al. (2018), we applied *k*‐means clustering to affluent flow and energy generation data. We evaluated alternative triggers and selected Affluent Flow based on vine‐copula dependence and statistical significance.

We estimate a spatial model of energy generation to capture hydrometeorological effects and interdependence among powerplants connected through the hydrological network.

### 3.1 Spatial Econometrics

Spatial econometrics models dependence among units connected through a spatial structure (Anselin 1988). In the present context, hydroelectric plants are connected through the hydrological network, making flow connectivity more relevant than geographic distance because plants in the same river system may be strongly related even when geographically distant. Plants along the same river system may be strongly related despite geographic distance, whereas nearby plants in different basins may interact only weakly.

For this reason, instead of relying on a conventional distance‐based weighting matrix, we construct a hydrological spatial matrix grounded in the river network. Let $W_{t}$ denote the $n_{t}\times n_{t}$ weighting matrix observed in period t, where $n_{t}$ is the number of active hydroelectric plants in that month. Each element $w_{ij,t}$ is binary and directional, indicating whether plant j is a first‐order hydrological neighbor of plant i in period t. Hence, $w_{ij,t}=1$ when a direct hydrological connection exists in the river network, and $w_{ij,t}=0$ otherwise. Since the matrix is binary, directional, and restricted to first‐order hydrological links, absolute row sums are uniformly bounded by one. Under the maintained hydrological network structure, column sums also remain bounded, so the resulting sequence of weighting matrices satisfies the standard boundedness conditions commonly used in spatial asymptotics. Because plants enter the sample over time, the hydrological network and spatial matrix vary by period. The panel is therefore unbalanced, as both the number of units and the spatial matrix vary over time.

Let $E_{t}$ denote the $n_{t}\times 1$ vector of electricity generation in period t, $X_{t}$ the $n_{t}\times k$ matrix of observed covariates, and $W_{t}$ the corresponding hydrological weighting matrix defined over the set of active plants in that period. The empirical specification is written as a spatial autoregressive model for an unbalanced panel with unit and time effects:

(5) $$E_{t}=\rho W_{t}E_{t}+X_{t}\beta +\mu_{i}+\tau_{t}+u_{t},t=1,\text{…},T.$$

Here, ρ measures endogenous spatial interaction in electricity generation, $\mu_{i}$ captures plant‐specific fixed effects, $\tau_{t}$ captures common time effects, and $u_{t}$ is the structural disturbance. The vector $X_{t}$ includes reservoir water volume, water inflow, average precipitation over the previous 3 months, wind speed, relative humidity, and interaction terms among these hydrometeorological variables. The model is estimated on the observed unbalanced panel, avoiding information loss and network distortions.

Before estimation, we test for residual spatial dependence. We begin with a two‐way fixed‐effects model and compute diagnostics that account for the fact that the hydrological weighting matrix varies over time. Moran's *I* is computed using period‐specific matrix $W_{t}$ and then aggregated across the panel to assess residual spatial autocorrelation. We also implement LM diagnostics for spatial lag and error dependence using period‐specific score contributions, making the tests compatible with a changing number of plants and a time‐varying spatial matrix. Because the hydrological network is directional, we further decompose each matrix as

(6) $$W_{t}=W_{s,t}+W_{a,t,}$$

where $W_{s,t}$ is the symmetric component and $W_{a,t}$ is the antisymmetric component:

(7)

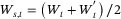



(8)

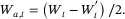




Following Chagas (2026), we then compute decomposed score tests to distinguish between contextual and directional sources of network dependence. The joint statistic $LM_{s,a}$ tests for residual dependence through either the symmetric or the directional component. The conditional statistic $LM_{s|a}$ tests whether the symmetric component remains relevant after controlling for the directionality, while $LM_{a|s}$ assesses whether the directional component adds information beyond symmetric exposure. Classical quadratic diagnostics (e.g., Moran's I and LM‐error) primarily capture the symmetric component of asymmetric networks, whereas the decomposed LM tests assess whether upstream–downstream orientation adds information beyond symmetric hydrological exposure.

The diagnostics confirm significant spatial dependence, supporting an asymmetric SAR specification. However, because the panel is unbalanced and the spatial matrix varies over time, standard maximum‐likelihood procedures are inappropriate. We therefore estimate the model by instrumental variables generalized method of moments (IV‐GMM), following the logic of Kelejian and Prucha (1998, 1999)’s spatial GMM‐IV approach, adapted to a two‐way fixed‐effects unbalanced panel with period‐specific matrices. The endogenous regressor $W_{t}E_{t}$ is instrumented with $X_{t}$, $W_{t}X_{t}$, and $W_{t}^{2}X_{t}$. Fixed effects are removed prior to IV‐GMM estimation with a HAC weighting matrix based on the period‐specific moments:

(9)

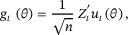

where $Z_{t}$ denotes the instrument matrix in period t. This accommodates heteroskedasticity and temporal dependence, in the spirit of Kelejian and Prucha (2007, 2010). After comparing alternative instrument sets and HAC bandwidths, we retain the two‐way fixed‐effects SAR‐IV‐GMM‐HAC specification. Additional details on hydrological network construction, unbalanced‐panel procedures, spatial diagnostics, IV‐GMM‐HAC estimation, robustness analyses, and impact calculations are provided in the Supplementary Appendix I.

### 3.2 Copulas

The use of regression analysis introduces some restrictive assumptions on the joint distribution of the variables employed in the model. Particularly, regression models limit the scope of the analysis to a linear correlation dependence structure, which is inadequate for representing dependency in the tails of multivariate distributions, jeopardizing the assessment of extreme losses (Bokusheva 2018).

We employ vine copulas as a complementary robustness tool to characterize the dependence structure among climatic variables, hydrological flow, and electricity generation. This analysis helps assess whether affluent flow provides a suitable trigger variable for the proposed insurance design and clarifies how dependence patterns differ across types of hydroelectric powerplants.

A copula links marginal distributions to a joint distribution of random variables. A copula $C\left(u\right)=C\left(u_{1},\;.\;.\;.,\;u_{d}\right)$ is a multivariate distribution function on ${\left[0,1\right]}^{d}$ with standard uniform marginal distributions (McNeil et al. 2005).

According to Sklar's theorem, if *F* is a joint distribution function with marginal distributions $F_{1},\;.\;.\;.,\;F_{d}$, then there exists a copula *C* such that for all $\left(x_{1},\;.\;.\;.,\;x_{d}\right)$:

(10) $$F\;\left(x_{1},\;.\;.\;.,\;x_{d}\right)=C\left(F_{1}\left(x_{1}\right),\;.\;.\;.,\;F_{d}\left(x_{d}\right)\right).$$

Also, any joint probability density function $f(x_{1},\;x_{2})$ can be expressed as (Czado 2019):

(11) $$f\;\left(x_{1},\;x_{2}\right)=c_{12}\;\left(F_{1}\left(x_{1}\right),\;F_{2}\left(x_{2}\right)\right)\cdot f_{1}\left(x_{1}\right)\cdot f_{2}\left(x_{2}\right),$$

and any conditional density $f(x_{1}|x_{2})$ can be expressed as (Czado 2019):

(12) $$f\;\left(x_{1}|x_{2}\right)=c_{12}\;\left(F_{1}\left(x_{1}\right),\;F_{2}\left(x_{2}\right)\right)\cdot f_{1}\left(x_{1}\right),$$

where *c* is the copula density and *f* and *F_i_* denote the probability density and cumulative marginal distribution functions, respectively.

There are many different families of copulas: elliptical (e.g., Gaussian/*t*‐Student), Archimedean (e.g., Clayton/Joe), extreme‐value (e.g., Tawn/Galambos), and so forth. Once the copula has been chosen, its parameters can be estimated using the maximum log‐likelihood method (Bokusheva 2018; Czado 2019).

However, the likelihood method assumes iid data, which may not hold in our case, as the data arise from time series. So, it is first necessary to apply an ARIMA‐GARCH filter to remove temporal dependence and thereby capture the dependence structure among the resulting residuals.

When d>2, many of *d*‐dimensional copulas do not have an explicit formula. To get through this, we use a vine‐copula structure, where the joint probability density function $f(x_{1},\text{…},x_{d})$ is rewritten using conditional densities as

(13) $$\begin{array}{ccc} f\;\left(x_{1},\text{…},x_{d}\right) & = & f_{d|1\text{…}\left(d-1\right)}\;\left(x_{d}|x_{1},\text{…},x_{d-1}\right)\\ & & \cdot \;f_{d-1|1\text{…}\left(d-2\right)}\left(x_{d-1\;}|x_{1},\text{…},x_{d-2}\right)\cdot \text{…}\cdot f_{1}\left(x_{1}\right). \end{array}$$

From Equation (12), each conditional density can be expressed in terms of a copula density *c* so Equation (13) can be rewritten as:

(14) $$\begin{array}{ccc} f\;\left(x_{1},\text{…},x_{d}\right) & = & \prod_{j=1}^{d-1}\prod_{i=1}^{d-j}c_{i,\left(i+j\right)|\left(i+1\right),\text{…},\left(i+j-1\right)}\\ & & \left(F\left(x_{i}|x_{i+1},\text{…},x_{i+j-1}\right),F(x_{i+j}|x_{i+1},x_{i+j-1})\right)\prod_{k=1}^{d}f_{k}\left(x_{k}\right).\; \end{array}$$

Vine copulas can be used to access causal dependence relationships. According Kiiveri et al. (1984), any joint probability density consistent with a causal relationship can be decomposed according to the underlying network structure. For instance, in a network of the form *X*_1_ → *X*_2_ → *X*_3_, the joint density can be expressed as

(15) $$f\;\left(x_{1},x_{2},x_{3}\right)=f\left(x_{1}\right)\cdot f\left(x_{2}|x_{1}\right)\cdot f\left(x_{3}|x_{2}\right)\;$$

and, consequently, conditional independence constraints arise directly from this factorization.

Using Equation (12), this joint density can be written in terms of the copulas $c_{12}$ and $c_{32}$. A notable case occurs $c(X_{1},X_{2}|X_{3})$ correspond to the independence copula: this indicates that given $X_{3}$, the variable $X_{2}$ is exogenous to it and exhibits no correlation with $X_{1}$. Namely, or $X_{2}$ is explanatory to $X_{3}$, or variations in $X_{2}$ have no direct impact on $X_{1}$.

Once we had modeled the variables’ causal dependence structure throughout the vine copulas, we are able to assess the joint probability of occurrence of the variables. The variable(s) directly related to the energy generation are suited to act as index(es) for the PI.

## 4 Results

### 4.1 Data and Exploratory Analyses

We gathered the data for Brazilian powerplants from the ONS official database, and the climate measures from the INMET. After treatment, the databases comprehend the generated energy by hydroelectric powerplants (in MWmed), their location and identification variables (e.g., name and hydrographic basin), alongside with a dummy variable indicating if it operates as run‐of‐river or water storage, and quantitative variables regarding water volume in the reservoirs (meters), water flow (m^3^/s), and meteorological data, such as precipitation levels (mm), temperature (°C), wind speed (m/s), and air relative humidity (%).

**FIGURE 1:**
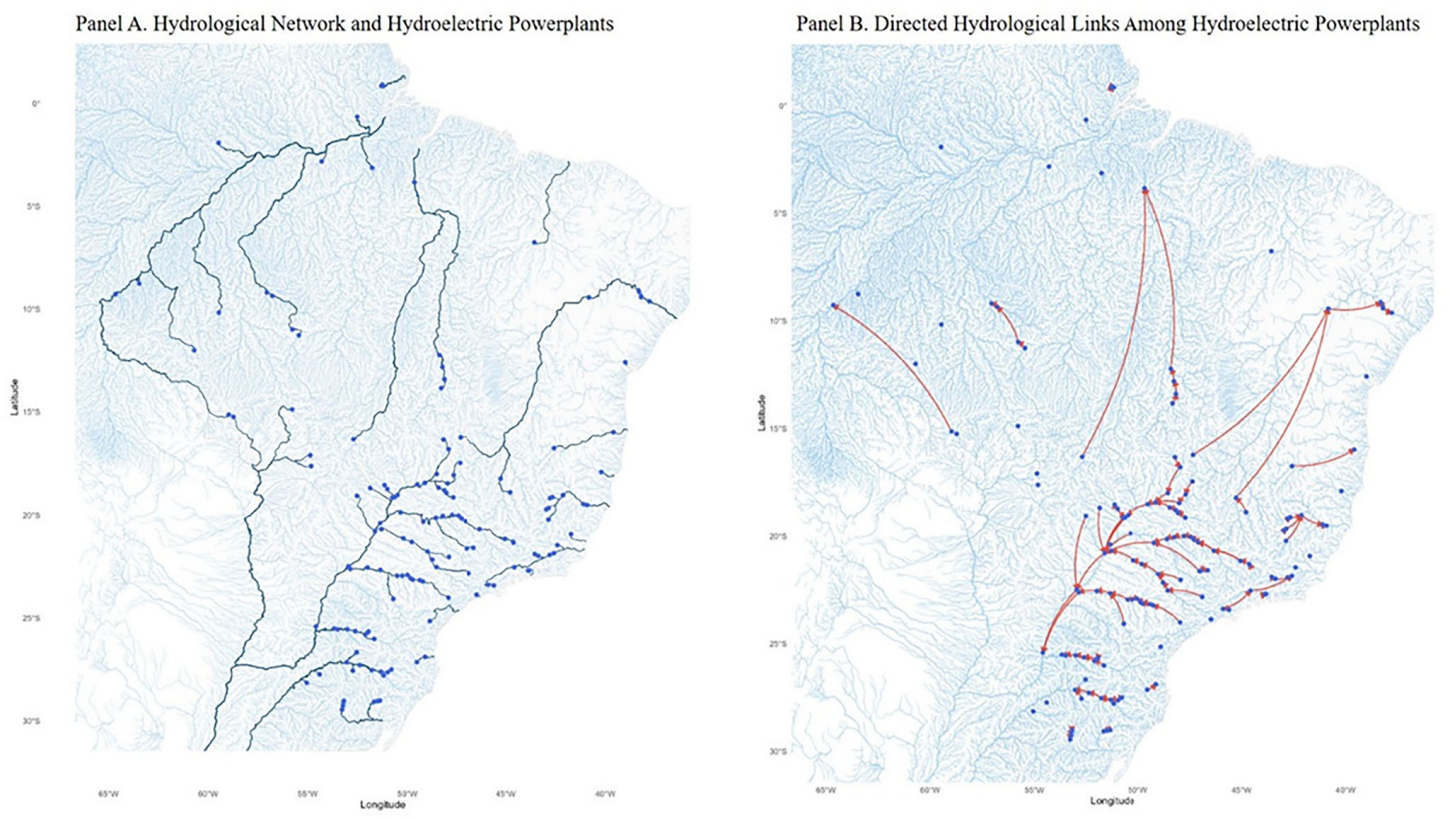
Hydrological network and spatial connectivity among Brazilian hydroelectric powerplants. *Note*: Panel A displays the river network geometry from HydroRIVERS and the georeferenced hydroelectric powerplants used in the empirical analysis. Panel B represents the directed upstream–downstream plant connections that define the neighborhood structure of the spatial weights matrix $W_{t}$. These links are constructed to reflect hydrological, rather than Euclidean, proximity.

We also utilized the HydroSHEDS framework^7^ (which provide globally consistent hydrographic layers) to construct the hydrological network underlying the spatial weighting matrices, combining the georeferenced powerplant data to hierarchical basin structure and accounting for upstream–downstream connectivity. Figure 1 illustrates these relations.

**FIGURE 2:**
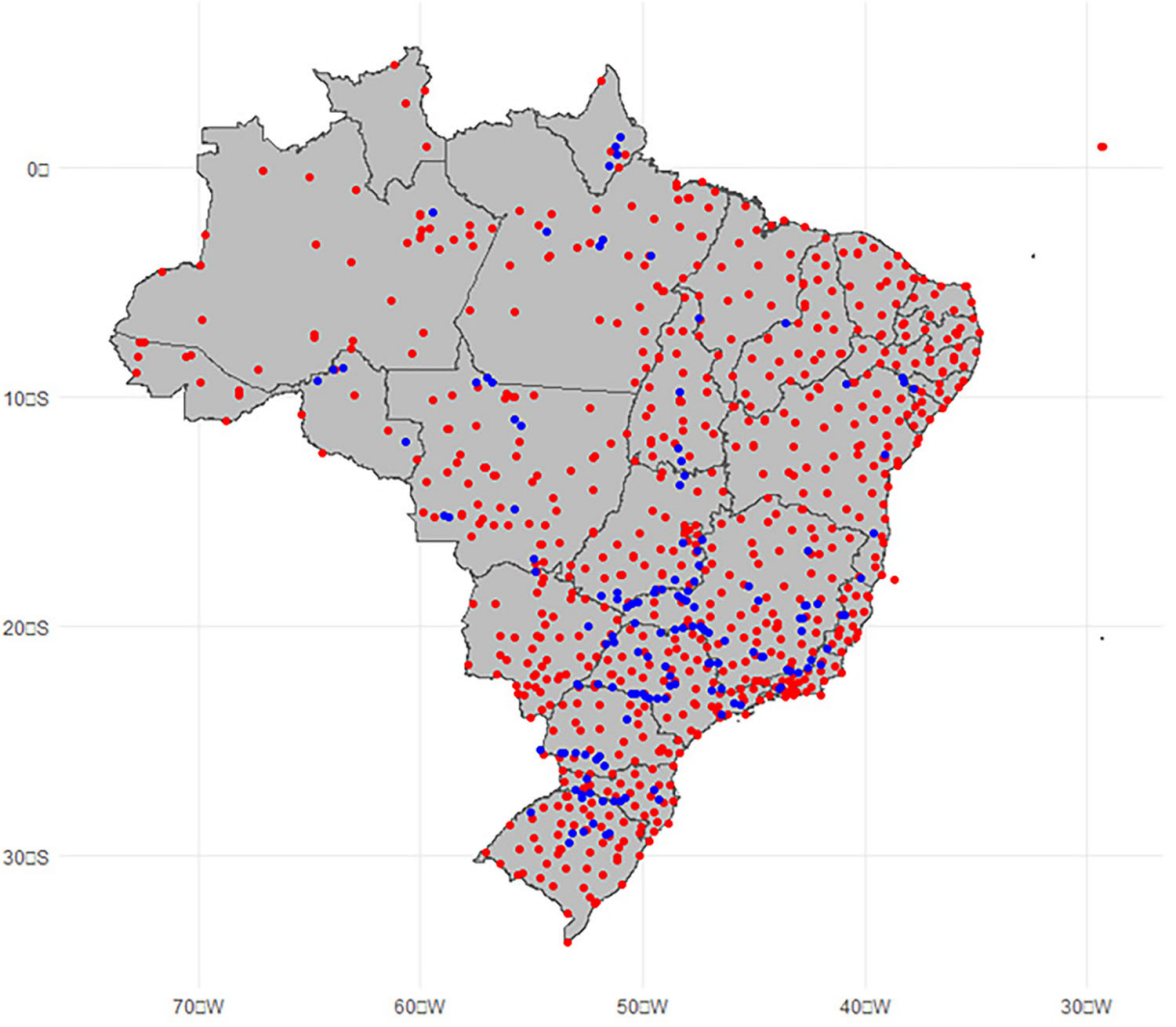
Brazilian hydro powerplants (blue) and meteorological meters (red).

**FIGURE 3:**
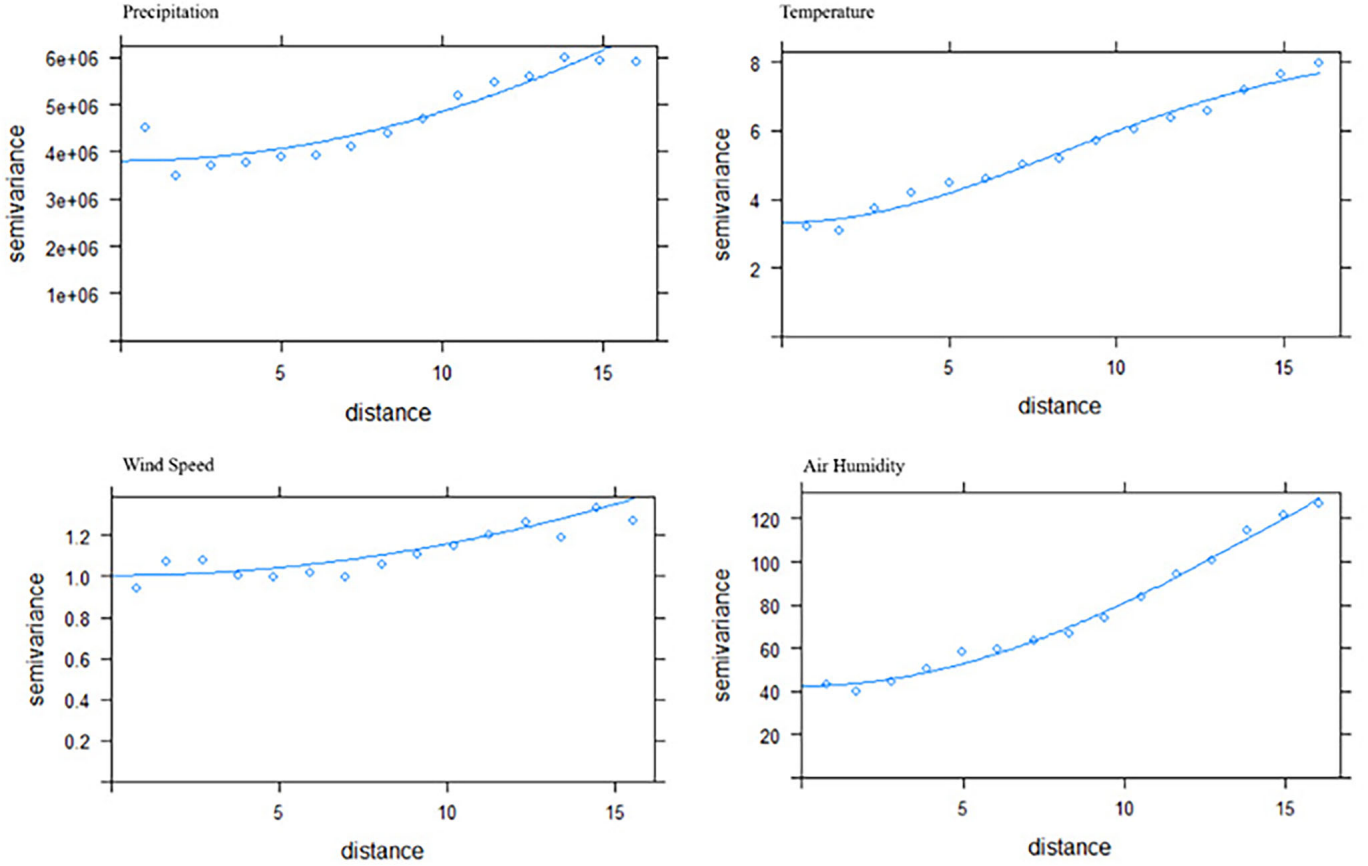
Meteorological data: Original and krigged.

**FIGURE 4:**
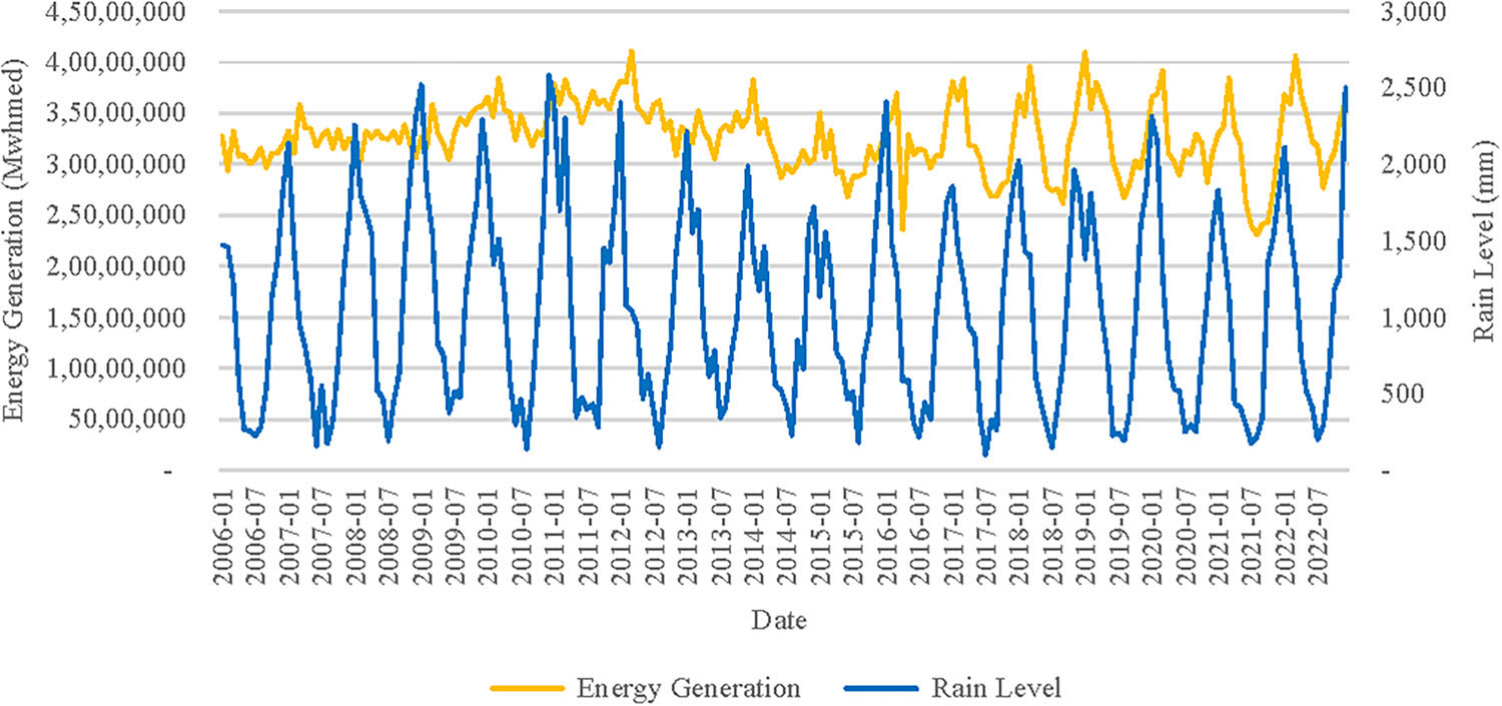
Generation × Precipitation in Brazil. *Note*: The energy generation series is the sum of all energy generated in each month by all the powerplants. The precipitation series is the average precipitation in each month considering all regions.

**FIGURE 5:**
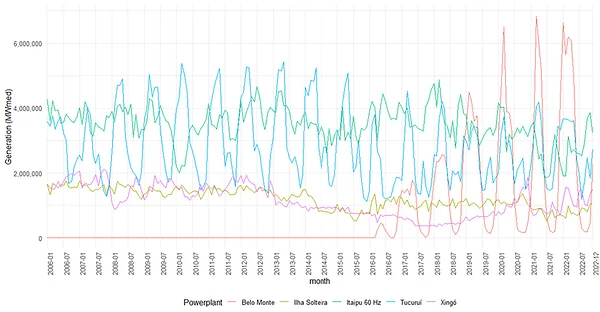
Top‐5 powerplants energy generation time series.

The data set is structured as an unbalanced monthly panel spanning from January/2006 to December/2022 and encompassing 151 powerplants. To ensure that we did not lose observations during the model estimation, we treated powerplants with null volume, particularly those categorized as run‐of‐river, setting their volume to zero. Tables 1, 2 and Figures 2, 3, 4, 5 present descriptive statistics.

**TABLE 1:** Powerplants by electricity subsystems.

Region	Number of powerplants
North (N)	10
Northeast (NE)	08
South (S)	30
Southeast (SE)	103
Total	151

**TABLE 2:** Ten largest powerplants by monthly average energy generation and type.

	Run‐of‐river				Storage water volume			
Ranking	Powerplant's name	State	Basin's name	Generation (Mwmed)	Powerplant's name	State	Basin's name	Generation (Mwmed)
1	Itaipu 60 HZ	PR	Paraná	3,490,762.0	Tucuruí	PA	Tocantins	2,847,237.8
2	Itaipu 50 HZ	—	Paraná	2,949,235.0	Ilha Solteira	SP	Paraná	1,203,226.2
3	Belo Monte	PA	Amazonas	1,888,693.7	São Simão	MG	Paranaíba	841,826.4
4	Xingó	AL	São Francisco	1,144,363.1	Salto Santiago	PR	Iguaçu	574,044.3
5	Santo Antônio	RO	Amazonas	1,071,824.2	Itumbiara	MG	Paranaíba	537,399.8
6	Jirau	RO	Amazonas	1,051,988.1	Água Vermelha	SP	Grande	524,462.0
7	Paulo Afonso IV	BA	São Francisco	831,938.8	Gov.Ney Braga	PR	Iguaçu	510,703.7
8	Porto Primavera	SP	Paraná	766,231.7	Marimbondo	MG	Grande	469,828.9
9	Jupiá	SP	Paraná	677,780.2	Gov.Bento Munhoz	PR	Iguaçu	463,135.1
10	Itá	SC	Uruguai	573,442.4	Luiz Gonzaga	PE	São Francisco	462,178.7

*Note*: Itaipu 60 Hz represents the Brazilian portion of Itaipu, while Itaipu 50 Hz represents the Paraguayan portion. These averages only consider the periods when the powerplant was operational (nonnull generation).

Figure 2 illustrates that there are sufficient meteorological meters close to each powerplant to enable obtaining accurate precipitation measurements. To assign the corresponding meteorological measures to each powerplant, we employed a kriging technique, based on the three nearest weather stations. This choice was motivated by computational efficiency and by the relatively close proximity among most meteorological stations. The procedure was performed separately for each month and then organized into the monthly panel structure. As an example of the procedure, Figure 3 presents the interpolated data for December/2022. Overall, the comparison between observed and interpolated values showed similar distributions, supporting the use of the krigged meteorological data set in the empirical analysis. Kriging harmonizes plant‐level weather covariates rather than generating identifying variation.

A powerplant's location does not necessarily where the generated energy is consumed. Powerplants located in the northern regions of Brazil, for example, may produce electricity that is primarily directed to the densely populated southeast, which serves as the country's residential and industrial hub. This is made possible by Brazil's well‐developed electrical network infrastructure, which includes extensive long‐distance transmission powerlines (Cataia 2019). Table 1 shows the number of powerplants present in each electricity subsystem.

Table 2 highlights the largest generators, which include both run‐of‐river and water‐storage plants and are concentrated in major Brazilian river basins. This reinforces the need to distinguish powerplant types while accounting for the hydrological structure underlying electricity generation in the modeling process.

Figure 4 illustrates the relationship between aggregate hydroelectric generation and precipitation in Brazil over time. Although precipitation is not the trigger in the proposed PI scheme, it drives hydrological conditions, explaining variations in affluent flow. The series move together, with generation increasing in wetter periods and declining during droughts.

The upward generation trend until mid‐2012 was disrupted by the 2014 hydrological crisis, which coincided with lower precipitation peaks and reduced generation. A similar pattern appears in 2021, when another drought episode reduced generation. Figure 4 also shows the seasonal pattern of precipitation, with higher levels during summer and lower levels during winter, consistent with Brazil's tropical and subtropical climates.

Figure 5 shows generation for the five largest powerplants, highlighting their contribution to aggregate hydroelectric production. Itaipu and Tucuruí dominate the initial period, while Belo Monte becomes increasingly relevant after becoming operational. This shift is accompanied by declining in production from Itaipu and Tucuruí, balancing supply.

### 4.2 The Spatial Model Estimation

We first estimate a two‐way fixed‐effects OLS model using the hydrometeorological variables described above. The vector includes reservoir volume, water inflow, 3‐month average precipitation, wind speed, relative humidity, and interaction terms. We then estimate a two‐way fixed‐effects SLX model with the same covariates augmented by their hydrological spatial lags. Finally, we estimate the preferred SAR‐IV‐GMM‐HAC specification, which incorporates endogenous spatial interaction through the hydrological network. The results are reported in Table 3.

**TABLE 3:** Results for different model structures estimation.

	OLS	SLX	SAR
Useful volume	1722.645^***^	1675.623^***^	1631.683^***^
	(63.939)	(63.826)	(29.802)
Affluent flow	56.485^***^	53.064^***^	59.854^***^
	(5.465)	(5.531)	(5.360)
Average 3‐month precipitation	18.916^***^	21.218^***^	12.354^***^
	(2.863)	(3.318)	(3.398)
Wind speed	−13,629.146^***^	−12,379.008^***^	−6668.346^***^
	(2652.658)	(3041.032)	(1.431)
Humidity	489.008^**^	123.229	486.106^***^
	(207.495)	(242.679)	(21.398)
Useful volume*Average 3‐month precipitation	−0.082^**^	−0.068	−0.094^**^
	(0.041)	(0.043)	(0.044)
Affluent flow*Average 3‐months precipitation	−0.006^***^	−0.005^***^	−0.008^***^
	(0.002)	(0.002)	(0.002)
Wind speed*Humidity	−2004.127^***^	−2203.262^***^	−1806.893^***^
	(241.233)	(283.849)	(13.974)
Lagged‐water volume		595.891^***^	
		(62.317)	
Lagged‐affluent flow		42.088^***^	
		(7.229)	
Lagged‐average 3‐month precipitation		−16.025^***^	
		(4.452)	
Lagged‐wind speed		2392.081	
		(4358.554)	
Lagged‐humidity		827.030^***^	
		(298.219)	
*Ρ*			0.322^***^
			(0.042)
*N*	152	152	152
*t*	204	204	204
AIC	732,466.6	732,234.8	
Moran‐I (*p*‐value)	0.1160	0.1062	
	(4.56e–45)	(4.41e–38)	
LM‐lag (*p*‐value)	197.164	103.225	
	(8.68e–45)	(2.99e–24)	
LM‐error (*p*‐value)	196.806	164.747	
	(1.04e–44)	(1.04e–37)	
LM_*s*,*a* (*p*‐value)	197.184	103.606	
	(1.52e–43)	(3.18e–23)	
LM_*s*|*a* (*p*‐value)	194.259	103.324	
	(3.74e–44)	(2.85e–24)	
LM_*a*|*s* (*p*‐value)	64.261	30.875	
	(1.09e–15)	(2.75e–08)	
*J*‐stat (*p*‐value)			20.609 (0.150)

*Note*: This table reports estimates for the baseline two‐way fixed‐effects OLS model, the corresponding SLX specification with hydrological spatial lags of the explanatory variables, and the preferred SAR model estimated by IV‐GMM with a HAC weighting matrix. Robust standard errors are reported in parentheses for coefficient estimates, while the values in parentheses reported below the diagnostic statistics are *p*‐values. *N* denotes the number of hydroelectric plants and the number of time periods used in each specification. For the OLS and SLX models, model fit is summarized by the Akaike Information Criterion (AIC). Moran's I reports the global residual spatial autocorrelation statistic computed on the stacked panel using the period‐specific hydrological weighting matrices. LM‐lag and LM‐error denote Lagrange Multiplier diagnostics for residual spatial lag and spatial error dependence, respectively. In asymmetric networks, Moran's I and LM‐error should be interpreted as diagnostics for the symmetric or contextual component of residual dependence, while directional relevance is assessed through the decomposed score tests proposed by Chagas (2026). For the SAR‐IV‐GMM‐HAC model, model adequacy is assessed through the overidentification *J*‐statistic and its *p*‐value. All specifications include plant and time fixed effects. Statistical significance is denoted by ***p < 0.001, **p < 0.01, *p < 0.05, and °*p* < 0.1. Variables are kept in levels rather than logarithms because several series contain zero values, and logarithmic transformations would therefore require additional treatment and reduce the direct interpretability of the coefficients.

Coefficients are broadly stable across models. Useful volume and affluent flow remain positive and statistically significant throughout, indicating that plants with greater reservoir storage and larger inflows generate more electricity. Average precipitation also enters positively in all cases, although its magnitude declines in the SAR specification. Interaction terms are consistently negative, suggesting diminishing marginal effects contribution among hydrometeorological variables when combined with higher levels of another.

Wind speed is negative and statistically significant in all three models, while humidity becomes clearly significant only in the SAR‐IV‐GMM‐HAC specification. This suggests that atmospheric conditions affect generation more complexly than rainfall alone would imply. The negative coefficient on wind speed should therefore be interpreted jointly with the humidity interaction term, since both variables are included mainly as controls for broader atmospheric conditions associated with precipitation dynamics.

The SLX estimates indicate that neighboring hydrological conditions matter, but not uniformly across variables. The spatial lags of useful volume, affluent flow, and precipitation are all statistically significant, showing that generation depends not only on local conditions but also on those observed at directly connected plants. By contrast, lagged wind speed and humidity coefficients are not significant, suggesting that the most relevant spillovers operate through hydrological rather than purely atmospheric channels.

Still, the comparison between OLS and SLX shows that spatially lagged covariates alone do not fully absorb spatial dependence. The preferred SAR‐IV‐GMM‐HAC model yields a positive and highly significant spatial autoregressive coefficient (*ρ* = 0.417), indicating that generation comoves across hydrologically connected plants even after controlling for observed local and neighboring covariates. The overidentification test does not reject the instrument set, so this specification is retained for the subsequent analyses.

Moran's I is reported in Table 3 as a global residual autocorrelation statistic for the stacked panel, using the period‐specific hydrological weighting matrices. The statistic remains positive and highly significant after both the OLS and SLX specifications. Its value declines only moderately, from 0.1160 in the OLS model to 0.1062 in the SLX model, indicating that the inclusion of spatially lagged covariates reduces, but does not eliminate, residual spatial dependence. The standard LM diagnostics lead to the same conclusion. Both LM‐lag and LM‐error strongly reject the null of no spatial dependence under the OLS and SLX null models. These results indicate that a purely nonspatial specification is inadequate and that spatial dependence remains a central feature of electricity generation even after controlling for observed local and neighboring hydrometeorological conditions.

For an asymmetric hydrological network, however, Moran's I and LM‐error are quadratic diagnostics and are therefore insensitive to the antisymmetric component of the weighting matrix. They mainly capture the symmetric, or contextual, component of residual dependence. To assess whether network direction matters, we complement the classical diagnostics with decomposed score tests based on (Chagas 2026). The decomposed tests show that both components are relevant. The joint test $LM_{s,a}$ is strongly significant under both the OLS and SLX null specifications, with statistics of 197.184 and 103.606, respectively. The conditional symmetric statistic $LM_{s|a}$ is also highly significant in both cases, confirming that contextual hydrological exposure remains an important source of dependence. More importantly for the hydrological interpretation, $LM_{a|s}$ is statistically significant after both OLS and SLX, with values of 64.261 and 30.875, respectively. This indicates that upstream–downstream orientation contains incremental information beyond simple hydrological connectivity. Although the symmetric component explains most dependence, direction remains relevant. This supports the use of the original asymmetric hydrological matrix in the structural specification.

Given these findings, we estimate a SAR model by IV‐GMM with two‐way fixed effects and a HAC weighting matrix. We focus on the SAR specification because the objective is to parameterize propagation in electricity generation across hydrologically connected plants, rather than to treat spatial dependence solely as a nuisance component in the disturbance term. In this setting, the SAR model directly measures how generation responds across hydrologically connected plants. The preferred specification uses the full instrument set $\left[X_{t},\;W_{t}\;X_{t},\;W_{t}^{2}\;X_{t}\right]$ and automatic HAC bandwidth. The spatial autoregressive coefficient is positive and significant, ρ=0.322, indicating network propagation. The overidentification test does not reject the instrument set, with a *J*‐statistic of 20.609 and a *p*‐value of 0.150. Accordingly, the SAR‐IV‐GMM‐HAC specification is retained for the spatial impact analysis.

Table 4 reports robustness checks for the SAR‐IV‐GMM‐HAC specification under alternative instrument sets and HAC bandwidth choices. Across all specifications, the spatial autoregressive coefficient remains positive and significant, ranging from 0.270–0.352, confirming network propagation in hydroelectric generation. The preferred specification, based on the *X* + *WX* + *W* ^2^ *X* instrument set with the automatic HAC bandwidth, yields ρ=0.322. Results are stable across HAC bandwidth choices and support the *X* + *WX* + *W* ^2^ *X* specification as the most reliable instrument set. Overall, results support positive spatial propagation and favor the *X* + *WX* + *W* ^2^ *X* specification.

**TABLE 4:** Robustness of the SAR‐IV‐GMM‐HAC estimates to instrument sets and HAC bandwidth choices.

Specification	ρ	*J*‐stat	*df*	*p*‐value	HAC bw	Number of instruments	Number of regressors
*X* + *WX* | bw auto	0.270^***^ (0.047)	16.24	7	0.023	4	16	9
*X* + *WX* | bw small	0.282^***^ (0.044)	18.488	7	0.01	2	16	9
*X* + *WX* | bw large	0.276^***^ (0.048)	16.037	7	0.025	6	16	9
*X* + *WX* + *W* ^2^ *X* | bw auto	0.322^***^ (0.037)	20.608	15	0.15	4	24	9
*X* + *WX* + *W* ^2^ *X* | bw small	0.331^***^ (0.038)	23.586	15	0.072	2	24	9
*X* + *WX* + *W* ^2^ *X* | bw large	0.320^***^ (0.036)	19.514	15	0.191	6	24	9
Reduced | bw auto	0.352^***^ (0.105)	2.394	3	0.495	4	12	9
Reduced | bw small	0.347^***^ (0.094)	2.696	3	0.441	2	12	9
Reduced | bw large	0.352^***^ (0.109)	2.262	3	0.52	6	12	9

*Note*: This table reports robustness checks for the preferred SAR‐IV‐GMM‐HAC specification under alternative instrument sets and HAC bandwidth (bw) choices. The reported coefficient is the spatial autoregressive parameter *ρ*, with robust standard errors in parentheses. “*X* + *WX*” uses the exogenous variables and their first‐order hydrological spatial lags as instruments; “*X* + *WX* + *W* ^2^ *X*” additionally includes second‐order spatial lags; “Reduced” denotes a reduced instrument set selected on the basis of post‐fixed‐effects variation. The *J*‐statistic and its *p*‐value refer to the overidentification test. “*df*” denotes its degrees of freedom, “Number of instruments” the number of instruments retained in the specification, and “Number of regressors” the number of regressors in the structural equation. The HAC bandwidth (bw) is the truncation lag used in the HAC weighting matrix: “bw auto” refers to the automatic bandwidth selected by the rule adopted in the paper, while “bw small” and “bw large” denote, respectively, smaller and larger bandwidth choices used for sensitivity analysis. Statistical significance is denoted by ***p < 0.001, **p < 0.01, *p < 0.05, and °*p* < 0.1.

Table 5 reports the average direct, indirect, and total effects implied by the preferred SAR‐IV‐GMM‐HAC specification. Hydrological conditions affect electricity generation both locally and through network spillovers. Useful volume, affluent flow, and average precipitation have positive direct effects, confirming the importance of local water availability. Positive indirect effects indicate that hydrological shocks propagate through the river network. Total effects exceed direct effects for all variables, consistent with network amplification. Negative interaction terms suggest diminishing marginal effects of water‐related variables at higher availability levels. Wind‐related effects are negative and should be interpreted primarily as controls for broader atmospheric conditions. Overall, generation risk is not purely plant‐specific, as hydrological shocks propagate across connected plants.

**TABLE 5:** Direct, indirect, and total effects from the preferred SAR‐IV‐GMM‐HAC specification.

Variable	Direct	Indirect	Total
Useful volume	1631.683^***^ (29.802)	316.659^***^ (52.951)	1948.342^***^ (62.922)
Affluent flow	59.854^***^ (5.360)	11.616^***^ (1.621)	71.470^***^ (5.582)
Average 3‐month precipitation	12.354^***^ (3.398)	2.398^***^ (0.758)	14.752^***^ (4.062)
Wind speed	−6668.346^***^ (1.431)	−1294.118^***^ (215.695)	−7962.464^***^ (215.726)
Humidity	486.106^***^ (21.398)	94.338^***^ (16.355)	580.443^***^ (30.309)
Useful volume * Average 3‐month’ precipitation	−0.094^**^ (0.044)	−0.018^*^ (0.009)	−0.112^**^ (0.053)
Affluent flow * Average 3‐month precipitation	−0.008^***^ (0.002)	−0.002^***^ (0.000)	−0.009^***^ (0.002)
Wind speed * Humidity	−1806.893^***^ (13.974)	−350.662^***^ (58.481)	−2157.555^***^ (60.623)

*Note*: This table reports the average direct, indirect, and total effects implied by the preferred SAR‐IV‐GMM‐HAC specification. Direct effects measure the average impact of a change in a covariate on electricity generation at the same hydroelectric plant. Indirect effects measure average spillover effects transmitted through the hydrological network, while total effects are the sum of direct and indirect effects. Because the model is spatial autoregressive, coefficient estimates cannot be interpreted directly as marginal effects; the effects reported here are obtained from the implied impact matrix of the SAR specification. Standard errors, shown in parentheses, are computed using the delta method based on the estimated variance–covariance matrix of the SAR‐IV‐GMM‐HAC model. Statistical significance is denoted by *** p<0.001, ** p<0.01, * p<0.05, and 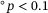.

### 4.3 Assessing the Interdependence Among the Variables Through Copulas

To validate the trigger choice and assess dependence among variables, we conducted a vine‐copula analysis on ARIMA‐GARCH filtered residuals (see the Supplementary Appendix II‐A). Analyses were conducted separately for run‐of‐river and water‐storage powerplants.

#### 4.3.1 Run‐of‐River Powerplants

Table 6 presents the best‐fitting vine copula for Itaipu, used as a representative run‐of‐river case because results were highly similar across plants (see the Supplementary Appendix II‐B). Table 6 also reports the best‐fitting copula (via maximum log‐likelihood estimation) for each edge of the vine structure. The notation (a,b|c,d) indicates that variables a,b are conditioned on variables c,d.

**TABLE 6:** Itaipu's vine copula.

		Copula		Dependence measures		
Tree	Edge	Family	Parameters	Kendall's tau	Upper tail	Lower tail
1	(1,2)	SurvGumbel	1.26	0.20		0.26
	(3,2)	SurvClayton	0.55	0.22	0.29	
	(6,4)	Gumbel90^*^	−1.43	−0.30		
	(6,3)	Gaussian	0.56	0.38		
	(6,5)	Gaussian	−0.25	−0.16		
2	(1,3|2)	Independent	—	0.00		
	(6,2|3)	Tawn	1.54/0.21	0.12	0.15	
	(4,3|6)	Independent	—	0.00		
	(5,3|6)	Independent	—	0.00		
3	(1,6|3,2)	Independent	—	0.00		
	(5,2|6,3)	Independent	—	0.00		
	(4,5|6,3)	Independent	—	0.00		
4	(1,5|6,3,2)	Independent	—	0.00		
	(4,2|5,6,3)	Independent	—	0.00		
5	(1,4|5,6,3,2)	Independent	—	0.00		

*Note*: *means the copula is rotated to the degrees presented in the family's name. The variables are: (1) Energy generation, (2) Affluent flow, (3) Average precipitation (Last 3 Months), (4) Temperature, (5) Wind speed, and (6) Humidity.

In Tree 1, the strongest correlation (0.38) is observed between precipitation and humidity. A lower correlation level between energy generation and affluent flow is noticeable (0.20), indicating that flow values correlate with generation values, which is consistent with the proposed model.

In the subsequent trees, we highlighted specific cells. They reveal that: (i) given the affluent flow, precipitation is exogenous to it and exhibits no correlation with energy generation. This suggests that either precipitation influences the affluent flow value, or variations in precipitation have no direct impact on the energy generation processes; (ii) humidity is exogenous to affluent flow and precipitation, and it does not correlate with energy generation. Namely, wind speed is an explanatory variable for changes in affluent flow and/or in precipitation but does not directly explain the variation in energy generation; (iii) wind speed is exogenous to affluent flow, precipitation, and humidity, and it is also not correlated with energy generation; and (iv) temperature is exogenous to affluent flow, precipitation, humidity, and wind speed, and it is also not correlated with energy generation.

**FIGURE 6:**
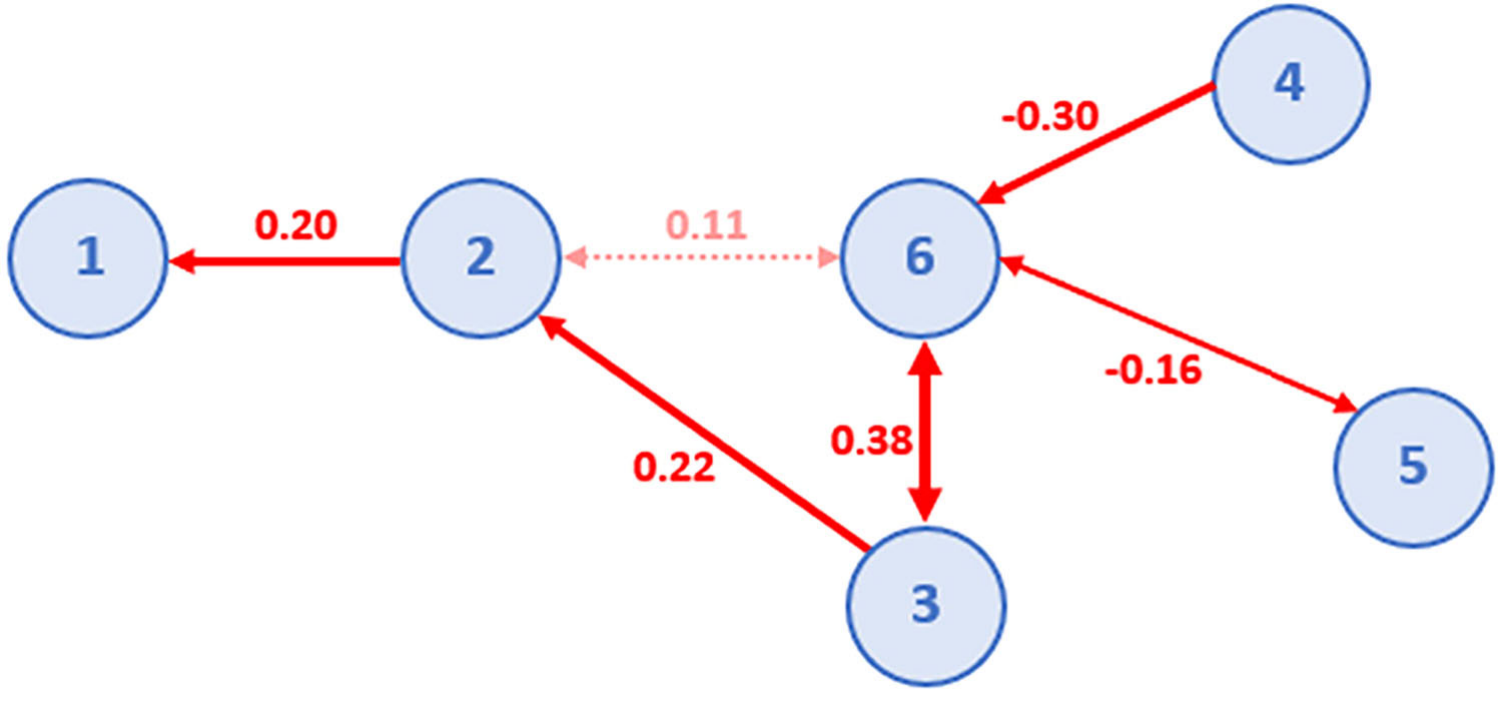
Network representing Itaipu's vine copula.

Figure 6 summarizes these findings in the network structure. The direction of each arrow indicates the causal inference, while its width represents the strength of dependence (indicated by the respective Kendall's tau).

In Figure 6, arrows in solid lines indicate the unconditional dependency relationship, while dotted lines indicate conditional dependencies. It becomes evident that meteorological factors are clearly associated, but the nature of this association is diffuse sometimes. Also, these meteorological factors collectively impact the affluent flow which, in turn, influence variations in energy generation. Thus, understanding the relationship between the pair (1,2) is fundamental to derive the required quantiles to price the insurance.

These findings align with the estimated spatial econometrics model. Temperature is exogenous to the model and was not included in SARAR. Affluent Flow exhibits the highest correlation with energy generation and a clear causal path, pointing to the use of this variable as a trigger for the run‐of‐river powerplants.

#### 4.3.2 Water‐Storage Powerplants

**FIGURE 7:**
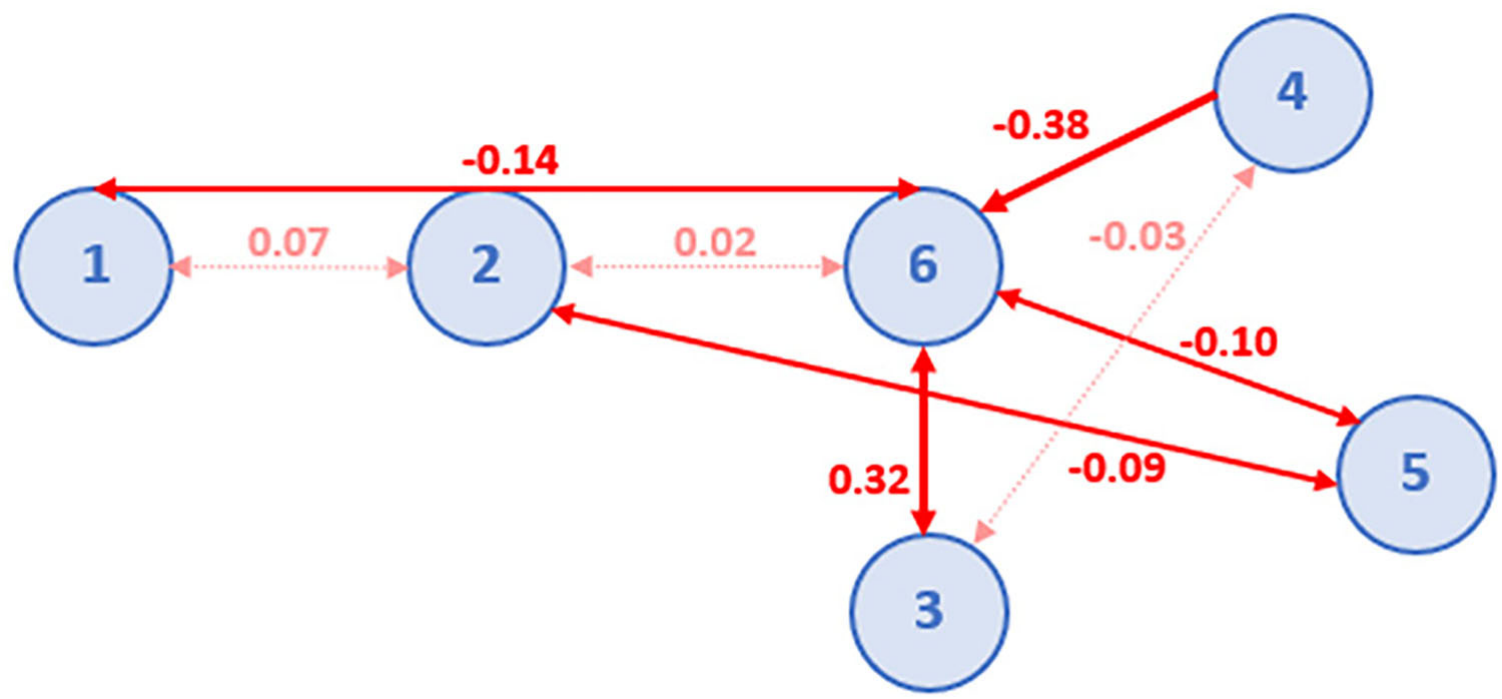
Network representing Ilha Solteira's vine copula.

**FIGURE 8:**
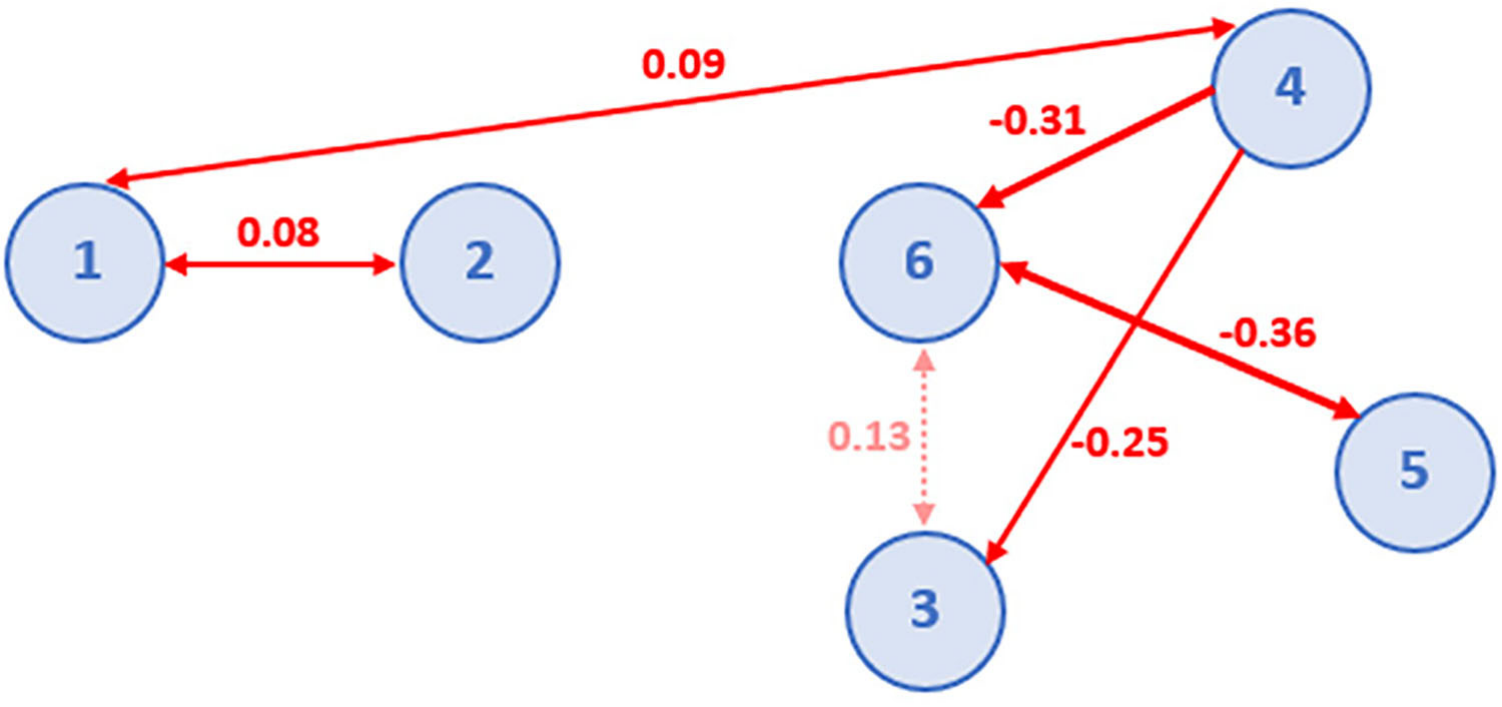
Network representing Tucuruí’s vine copula.

The dependence structure results for individual water‐storage powerplants were also remarkably similar among themselves (see the Supplementary Appendix II‐C), so we will only present Ilha Solteira and Tucuruí as reference cases (Tables 7 and 8, and Figures 7 and 8).

**TABLE 7:** Ilha Solteira's vine copula.

		Copula		Dependence measures		
Tree	Edge	Family	Parameters	Kendall's tau	Upper tail	Lower tail
1	(1,6)	Frank	−1.27	−0.14		
	(2,5)	Clayton90^*^	−0.21	−0.09		
	(6,5)	Gumbel270^*^	−1.12	−0.10		
	(6,3)	Frank	3.14	0.32		
	(6,4)	Gumbel90^*^	−1.61	−0.38		
2	(1,4|6)	Independent	—	0.00		
	(2,6|5)	Tawn180^*^	7.50/0.02	0.02		0.02
	(3,4|6)	Tawn270^*^	−9.91/0.03	−0.03		
	(4,5|6)	Independent	—	0.00		
3	(1,5|4,6)	Independent	—	0.00		
	(2,4|6,5)	Independent	—	0.00		
	(3,5|6,4)	Independent	—	0.00		
4	(1,2|5,4,6)	Joe	1.13	0.07	0.16	
	(2,3|4,5,6)	Independent	—	0.00		
5	(1,3|2,5,4,6)	Independent	—	0.00		

*Note*: *means the copula is rotated to the degrees presented in the family's name. The variables are: (1) Energy generation, (2) Useful volume, (3) Average precipitation (Last 3 months), (4) Temperature, (5) Wind speed, and (6) Humidity.

**TABLE 8:** Tucuruí’s vine copula.

		Copula		Dependence measures		
Tree	Edge	Family	Parameters	Kendall's tau	Upper tail	Lower tail
1	(1,2)	Clayton180^*^	0.17	0.08	0.02	
	(1,4)	Gumbel	1.10	0.09	0.12	
	(6,4)	Gumbel270^*^	−1.44	−0.31		
	(3,4)	Gumbel90^*^	−1.34	−0.25		
	(6,5)	Frank	−3.66	−0.36		
2	(4,2|1)	Independent	—	0.00		
	(6,1|4)	Independent	—	0.00		
	(3,6|4)	Clayton	0.29	0.13		0.09
	(5,4|6)	Independent	—	0.00		
3	(6,2|4,1)	Independent	—	0.00		
	(5,1|6,4)	Independent	—	0.00		
	(5,3|6,4)	Independent	—	0.00		
4	(5,2|6,4,1)	Independent	—	0.00		
	(3,1|5,6,4)	Independent	—	0.00		
5	(3,2|5,6,4,1)	Independent		0.00		

*Note*: *means the copula is rotated to the degrees presented in the family's name. The variables are: (1) Energy generation, (2) Useful volume, (3) Average precipitation (Last 3 months), (4) Temperature, (5) Wind speed, and (6) Humidity.

Several notable differences are observed in comparison with run‐of‐river powerplants. First, energy generation and water volume are not correlated unconditionally (e.g., Ilha Solteira) or the correlation, while still significant, is less strong (e.g., 0.08 for Tucuruí) as it was for energy generation and climate variables (e.g., temperature for Tucuruí). Second, the dependence observed in the lower tail between variables in run‐of‐river powerplants is no longer present for Tucuruí neither Ilha Solteira. In the latter case, there is no dependence in the lower tail between any two variables. Finally, the presence of conditional independence relationships given unconditional dependence, such as wind speed and temperature being independent given humidity (Ilha Solteira). However, there is no longer a structured dependence between wind and temperature unconditionally.

These findings suggest a more diffuse set of dependencies, making it challenging to infer causal relationships when constructing the network structure. The dependencies between variables in Ilha Solteira and Tucuruí exhibit a different pattern than those in run‐of‐river powerplants.

The same diffuse patterns are observed in the case of other water‐storage powerplants: or the water volume and energy generation are uncorrelated (unconditionally), or the dependence are bidirectional. The bidirectionality in the pair (1,2) can be due to the operational characteristics of water‐storage powerplants, especially human intervention. As the water flow that leaves the reservoirs can be controlled to meet the energy demand, at certain times the volume remaining in the reservoirs can be caused by the expected energy generation levels, while the amount of generated energy depends on the available water volume.

These diffuse relationships suggest that this study is limited to parameterizing insurance contracts exclusively for run‐of‐river powerplants, as there is no clear and well‐defined relationship between energy generation and other variables for water‐storage powerplants. Also, human managing could, in some cases, induce moral hazards, as the water volume could be intentionally put to a lower level to activate the insurance, while the energy generation would not immediately be jeopardized.

### 4.4 Defining the Insurance Trigger

Following Kusuma et al. (2018), we employ a clustering technique to identify instances in which both E and A were low levels. The chosen technique was the k‐means clustering, and, for each powerplant, we clustered the date into three groups (high/medium/low). This number was determined as optimal based on a scree‐plot analysis (see the Supplementary Appendix III‐A). We then computed different thresholds scenarios, assessing the lower cluster quantiles.

**FIGURE 9:**
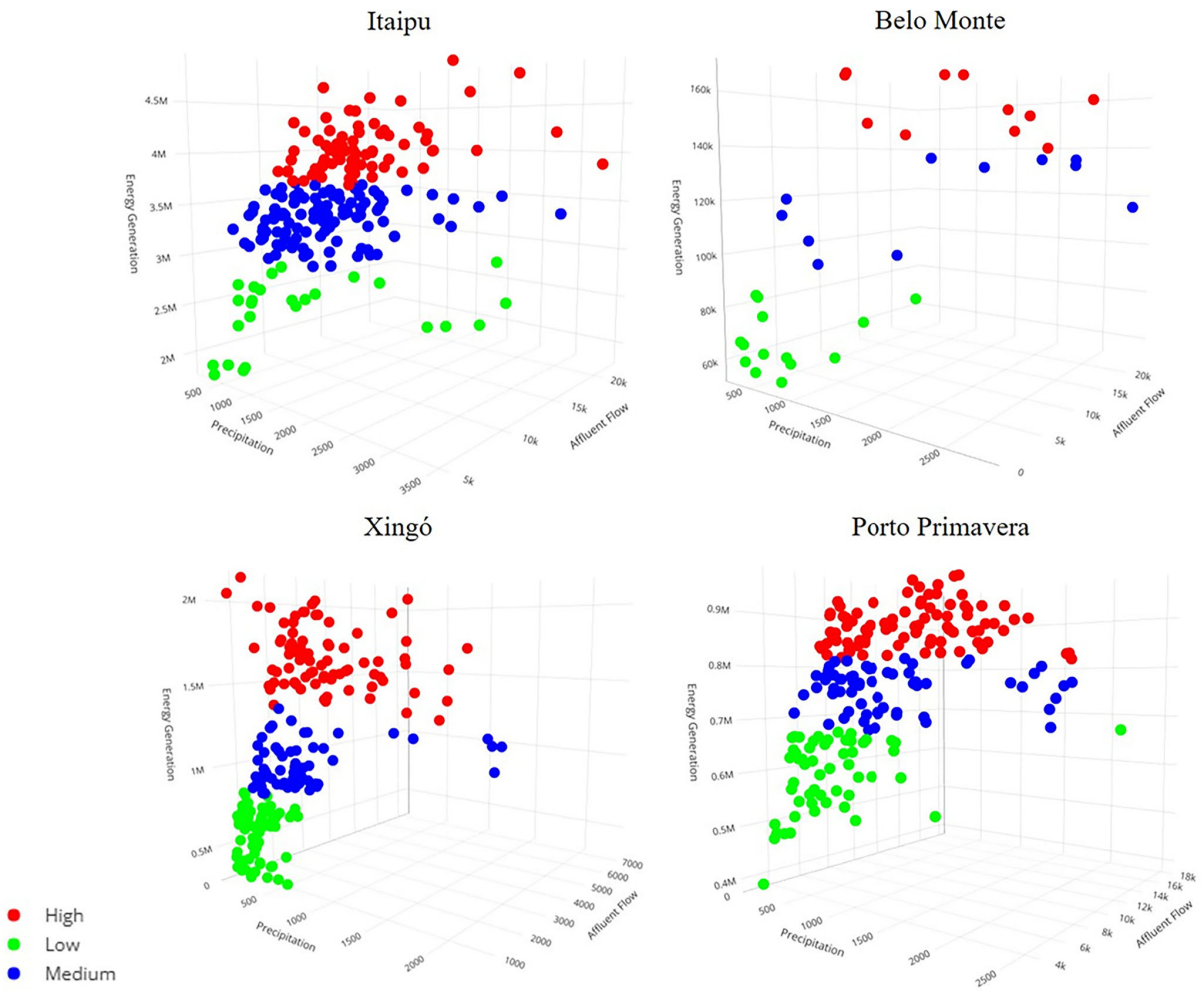
Clusters’ distributions. *Note*: For Belo Monte, we considered only the data from January/2020 onward, when the series gains stationarity, so that there is no bias from the early years when the energy generation levels were lower.

As each powerplant exhibits unique characteristics, we cannot define a one‐size‐fits‐all trigger that applies to all of them. Therefore, this aspect of the insurance contract must be tailored to each powerplant individually. We will present the calculations for the largest run‐of‐river energy generator of each region, hence: Itaipu (South), Belo Monte (North), Xingó (Northeast), and Porto Primavera (Southeast). Figure 9 shows the clustering distributions.

Table 9 presents possible trigger and exit levels for these powerplants, as quantiles calculated from the identified lower clusters.

**TABLE 9:** Trigger and exit thresholds.

	Trigger					Exit threshold		
Powerplant	Average	Median	35% Quantile	30% Quantile	First Quartile	5% Quantile	2.5% Quantile	1% Quantile
Itaipu	9160.68	7146.32	6282.79	6212.97	6074.92	5107.42	4849.49	4752.89
Belo Monte	1953.61	1227.92	888.15	855.60	846.51	787.33	736.11	705.38
Xingó	732.81	745.03	691.37	644.64	609.81	562.17	556.47	554.07
Porto Primavera	4845.22	4723.92	4479.60	4352.28	4213.87	3582.68	3538.91	3201.40

**TABLE 10:** Distributions and frequency probabilities.

Distributions with best fit		$F(A_{\mathrm{Tr}})$					$F(A_{E})$		
Powerplant	Distribution of *A*	Average	Median	35% Quantile	30% Quantile	First Quartile	5% Quantile	2.5% Quantile	1% Quantile
Itaipu	Gamma	0.3269	0.1017	0.0470	0.0437	0.0378	0.0110	0.0074	0.0063
Belo Monte	Student‐*t*	0.5607	0.3161	0.1295	0.1116	0.1067	0.0764	0.0533	0.0411
Xingó	Student‐*t*	0.1391	0.1523	0.0978	0.0589	0.0366	0.0158	0.0141	0.0134
Porto Primavera	Student‐*t*	0.1633	0.1346	0.0846	0.0634	0.0443	0.0037	0.0029	0.0003

To determine $F(A_{Tr})$ and $F(A_{E})$, we estimated Affluent Flow distributions for each powerplant and its value on the corresponding quantile from Table 10. The best‐fit results were chosen based on BIC. Further details on the fitting process, such as QQ‐plots, CDFs and parameters, are provided in the Supplementary Appendix III‐B.

### 4.5 Insurance Results

We assume a constant on‐grid energy price ($G_{w}$) of 292.74 BRL/MWh, corresponding to the 2026 energy generation tariff for residential consumers in Brazil.^8^ Since the tariff is stated in MWh, the MWmed energy generation data must be multiplied by the number of productive hours on each month to obtain the corresponding MWh measurement. We utilized the measure from the powerplants’ latest official reports. When unavailable, productive hours are assumed to equal 95% of total monthly hours.

Based on these premises and following Equations (1)–(4), we can compute the total indemnity amount that an insurance company would have paid if the powerplants had such contract throughout the analyzed period, as well as the received premiums. Table 11 shows an illustration of these calculations, specifically for Itaipu.

**TABLE 11:** Payout and premium calculation for Itaipu.

Month	Affluent flow	Bellow trigger?	Bellow exit?	Predicted “loss” of generation (Mwmed)	Productive hours	Payout (BRL)	Probability	Premium (BRL)
m	$A_{o}$	$A_{o}<A_{Tr}$	$A_{o}<\;A_{E}$	$\left(I_{w.m}\right)$	97.25%^9^ of the hours in the month	$S(I_{w.m})$	$P(I_{i})$	$S\left(I_{w.m}\right)\times P\left(I_{i}\right)$
2006–01	11,911.99	No	No		706.8		0.0396	
2006–02	11,030.25	No	No		638.4		0.0396	
……………………………………………………………………………………………………………………………………………………………………………………………………………………………………………….								
2021‐04	6238.02	Yes	No	2,679.60	700.2	549,255,922	0.0396	21,750,534
2021‐05	5869.04	Yes	No	24,764.53	723.54	5,245,353,771	0.0396	207,716,009
2021‐06	5704.03	Yes	No	34,641.18	700.2	7,100,629,668	0.0396	281,184,935
2021‐07	4688.48	Yes	Yes	85,788.74	723.54	18,170,835,394	0.0396	719,565,082
2021‐08	4946.10	Yes	No	80,006.44	723.54	16,946,091,243	0.0396	671,065,213
2022‐01	5664.86	Yes	No	36,985.52	723.54	7,833,870,600	0.0396	310,221,276
2022‐02	6037.25	Yes	No	14,696.27	653.52	2,811,565,272	0.0396	111,337,985
2022‐05	6187.92	Yes	No	5,678.23	723.54	1,202,701,447	0.0396	47,626,977
2022‐07	5591.40	Yes	No	41,382.21	723.54	8,765,127,805	0.0396	347,099,061

*Note*: Retrieved from Itaipu's 2022 Report, section *1.1.2 Manutenção ‐ Disponibilidade das unidades geradoras*: https://itaipu.gov.br/wp‐content/uploads/2024/12/RelAnual_2022_.pdf.

Table 11 uses Itaipu's lower cluster 35% quantile as trigger and the 2.5% quantile as exit threshold. This scenario was determined to be the most favorable in terms of feasibility for both the insurer and the powerplant. With these parameters, 4.4% of the observed months would have qualified for indemnification, a reasonable proportion for extreme‐losses insurance contracts. The total payout would have amounted to BRL 68,625,431,121, with the majority of claims concentrated in 2021, which was the most severe drought of Itaipu's history. Hence, the insurance would mitigate high‐cost systemic risks.

Given the substantial magnitude of the payouts, it becomes imperative for such an operation to be backed by reinsurance. The monthly actuarially fair premium would be:

(16) $$\begin{array}{ccc} P_{m} & & =\frac{1}{M}\;\sum_{m=1}^{M}P\left(I_{i}\right)\times S\left(I_{w,m}\right)=\frac{0.0396\times \mathrm{BRL}\;68,625,431,121}{204}\\ & & =\;\;\mathrm{BRL}\;13,321,407\; \end{array}$$

or BRL 159,856,867 annually. This value must be adjusted to account for the insurer's loadings, expenses and tax obligations, resulting in the actual commercial premium. Only to exemplify, if these loadings amounted to 70%, the resulting annual commercial premium would be BRL 271,756,707, which represents 2% of Itaipu's annual operational earnings,^10^^,^ 11 therefore making it a viable contract for both parties. It offers low frequency, high severity coverage with an affordable premium.^12^

Results for the other powerplants were computed with the same methodology and are summarized in Table 12. Detailed results considering other triggers and thresholds are available upon request.

**TABLE 12:** Insurance design results.

Powerplant	Chosen triggers (Quantiles)	Chosen exit thresholds (Quantiles)	Expected claims frequency ($P(I_{i})$)	Observed claims frequency	Annual payout (BRL)	Annual risk premium (BRL)	Payouts during droughts of 2014–2016 or 2021–2022?
Itaipu	35%	2.5%	4.0%	4.4%	4,036,790,066	159,856,887	Yes
Belo Monte	50%	2.5%	26.3%	3.4%	1,770,084,710	465,178,262	Yes
Xingó	25%	2.5%	2.3%	7.8%	276,025,339	6,210,570	No
Porto Primavera	25%	2.5%	4.1%	6.9%	3,529,706,275	146,129,840	Yes

*Note*: for Itaipu, we used a 97.25% proportion for operational hours and for Porto Primavera, 96.2%. For the other powerplants, this information was not available, so we used 95%.

https://www.itaipu.gov.br/sites/default/files/af_df/RelAnual_2022_.pdf.

https://api.mziq.com/mzfilemanager/v2/d/691c9da5‐45e0‐458f‐a3da‐41982b1730fc/79ed366b‐e250‐904d‐9948‐fd438d491ec2?origin=1.

The triggers were selected to standardize expected claims frequency level across powerplants, aiming at a 4% observed frequency (or as low as possible with the 25%‐quantile trigger, if higher than 4%), similarly to Itaipu and at an acceptable level to reinsurance underwriters, as this would typically be a tail risk.

Belo Monte presents the lowest claim frequency and is the only powerplant with observed claim frequencies way below the expected level because its historical series is dominated by drought periods, making trigger activation comparatively rare. Xingó and Porto Primavera, conversely, present higher frequencies than predicted, suggesting that their CDF models could be improved.

Viability also depends on loss ratios and portfolio composition. As with any insurance product, viability depends on risk pooling across the portfolio. Therefore, product viability depends not only on individual pricing but also on portfolio composition. Diversification across regions and reinsurance support are key mechanisms for managing loss ratios.

Table 12 suggests that PI mitigate drought‐related hydrological risk, although contract design must be tailored to powerplant characteristics. The magnitude of premiums and payouts also highlights the importance of reinsurance support.

Supplementary analyses for water‐storage plants (Appendix IV) reinforces the vine‐copula results, confirming that operational flexibility weakens the link between hydrological conditions and generation, increasing basis risk and reducing contract feasibility.

## 5 Concluding Remarks

This study developed a PI scheme for Brazilian hydroelectric generators exposed to hydrological and climate risk. The proposed product may help manage drought‐related generation losses in Brazil, where electricity production remains highly dependent on hydropower.

Our paper contributes by incorporating hydrological network dependence into PI pricing. The preferred SAR‐IV‐GMM‐HAC specification shows that electricity generation depends on both local hydrometeorological conditions and network spillovers across connected plants. Vine copulas results further support affluent flow as the trigger by showing its role in linking hydrometeorological conditions and electricity generation.

The insurance design results suggest that PI can mitigate drought‐related hydrological risk, especially for run‐of‐river plants. These plants provide a more direct link between affluent flow and generation losses, making them more suitable for index‐based coverage. By contrast, water‐storage plants involve more complex dependencies among inflows, reservoir management, and generation decisions, weakening the link between the trigger index and insured losses and raising additional moral hazard concerns.

Thresholds should therefore be defined at the powerplant level, reflecting each facility's generation profile and hydrological conditions. Given the magnitude of premiums and payouts, implementation would also likely require reinsurance support and portfolio diversification across plants and regions.

Future research could refine the estimation strategy, test alternative pricing and trigger structures, explore machine‐learning approaches, and extend the framework to other renewable energy sources or countries.

## Funding

João Vinícius de França Carvalho was supported by the National Council for Scientific and Technological Development (CNPq) under Grant [number 305589/2024‐5]. André Luis Squarize Chagas was supported by the National Council for Scientific and Technological Development (CNPq) under Grant [number 300732/2025‐2].

## Conflicts of Interest

The authors declare no conflicts of interest.

## Supporting information




**Supporting Information**: risa70347‐supp‐0001‐SuppMat.docx

**Supporting Information**: risa70347‐supp‐0002‐SuppMat.docx

**Supporting Information**: risa70347‐supp‐0003‐SuppMat.docx

**Supporting Information**: risa70347‐supp‐0004‐SuppMat.docx

## Data Availability

The data that support the findings of this study are available from the corresponding author upon reasonable request.

## Endnotes

1
https://statranker.org/economy/top‐10‐countries‐in‐electricity‐production‐for‐2025/.

2
https://www.ons.org.br/paginas/sobre‐o‐sin/o‐sistema‐em‐numeros.

3
https://statranker.org/economy/top‐10‐countries‐in‐electricity‐production‐for‐2025/.

4
https://www.power‐technology.com/features/featurethe‐worlds‐longest‐power‐transmission‐lines‐4167964/?cf‐view&cf‐closed.

5
https://www.enel.com/pt/learning‐hub/renovaveis/energia‐hidreletrica.

6
https://www.enel.com/pt/learning‐hub/renovaveis/energia‐hidreletrica.

7
https://www.hydrosheds.org/products.

8
https://www.enel.com.br/pt‐saopaulo/Para_Voce/tarifa‐energia‐eletrica.html.

9 Retrieved from Itaipu's 2022 Report, section 1.1.2 *Manutenção ‐ Disponibilidade das unidades geradoras*: https://itaipu.gov.br/wp‐content/uploads/2024/12/RelAnual_2022_.pdf.

10 Considering a BRL/USD = 5.00 exchange rate.

11
https://www.itaipu.gov.br/sites/default/files/af_df/RelAnual_2022_.pdf.

12 We intended to evaluate the proportion of the insurance premium over the operational revenue for other powerplants as well. However, this information was not available for other powerplants, as most are not publicly traded companies, and some are part of energetic conglomerates that only provide information for the Holding Company.
