# Supplementary material for: When There's No November Rain: Developing a Parametric Insurance for Hydroelectric Energy Generators in Brazil
Source: Risk Anal. 2026 Sep 3;46(9):e70347. doi: 10.1111/risa.70347 (PMC13542624; doi:10.1111/risa.70347)
Supplement: Supplementary file 1 [file RISA-46-0-s001.docx]

**Supplementary Appendix III – Clusters and Probability Distribution Fits**

**Appendix III-A. Cluster analyses**

Figure A.1 shows the scree plots, which indicate 3 levels are adequate for cluster differentiation.

**Figure A.1.** Scree Plots

Table A.1 summarizes the k-means clustering results. The high Between SS / Total SS values indicate that the three-cluster solution captures a substantial share of the variation in the variables used for clustering.

**Table A.1.** Cluster specifications

| **Powerplant** | **Cluster** | **n** | **Mean generation** | **Mean precipitation** | **Mean affluent flow** | **Between SS / Total SS** |
| --- | --- | --- | --- | --- | --- | --- |
| Itaipu | Low | 26 | 2,431,308 | 1,058.87 | 9,160.68 | 82.4% |
|  | Medium | 103 | 3,347,142 | 1,106.28 | 10,177.04 | 82.4% |
|  | High | 75 | 4,055,277 | 1,383.38 | 11,876.94 | 82.4% |
| Belo Monte | Low | 14 | 68,738 | 698.59 | 1,953.61 | 89.4% |
|  | Medium | 11 | 120,631 | 1,656.25 | 10,844.62 | 89.4% |
|  | High | 11 | 155,83 | 1,650.99 | 13,533.04 | 89.4% |
| Xingó | Low | 63 | 605,029 | 296.68 | 732.81 | 88.7% |
|  | Medium | 63 | 1,034,234 | 590.82 | 1,216.74 | 88.7% |
|  | High | 78 | 1,668,929 | 596.24 | 2,100.42 | 88.7% |
| Porto Primavera | Low | 54 | 600,177 | 601.73 | 4,845.22 | 85.4% |
|  | Medium | 63 | 758,255 | 995.15 | 6,478.68 | 85.4% |
|  | High | 87 | 875,077 | 1,031.92 | 7,828.09 | 85.4% |

**Appendix III-B. Triggers, Exits and Probability Fits**

Table B.1 reports the Bayesian Information Criterion (BIC) for each candidate distribution. Candidate distributions were fitted separately for each powerplant affluent flow data. The selected distribution corresponds to the lowest BIC among the candidate distributions.

The candidate distributions were selected because they represent a diverse set of distributional shapes commonly used to model environmental and hydrological variables, including light-tailed (Normal), skewed positive-support distributions (Exponential, Gamma, Weibull), and heavy-tailed alternatives (Student-t and Pareto). The objective was not to exhaustively evaluate all possible distributional families, but rather to compare a set of theoretically plausible specifications capable of capturing the asymmetry and tail behavior typically observed in hydrological flow series.

Distribution selection was based on the Bayesian Information Criterion (BIC). BIC was preferred because it balances model fit and parsimony, imposing a stronger penalty for model complexity than alternative information criteria such as AIC. Since the purpose of this stage was to identify a parsimonious distribution for the conversion of trigger and exit thresholds into cumulative probabilities, BIC provides an appropriate criterion for model comparison. The selected distribution for each powerplant corresponds to the lowest BIC among the candidate specifications.

**Table B.1.** BIC values for candidate distributions

| **Powerplant** | **Exponential BIC** | **Gamma BIC** | **Normal BIC** | **Weibull BIC** | **Student-t BIC** | **Pareto BIC** | **Selected distribution** |
| --- | --- | --- | --- | --- | --- | --- | --- |
| Itaipu | 4,197.691 | **3,820.134** | 3,847.297 | 3,863.304 | 3,835.392 | 9,840.464 | Gamma |
| Belo Monte | 724.514 | 728.546 | 754.095 | 728.071 | **715.972** | 1,565.512 | Student-t |
| Xingó | 3,370.466 | 3,244.752 | 3,352.323 | 3,270.919 | **3,186.388** | 3,384.327 | Student-t |
| Porto Primavera | 4,002.951 | 3,650.891 | 3,696.193 | 3,711.045 | **3,641.380** | 9,243.836 | Student-t |

Table B.2 reports the estimated parameters of the selected distribution for each powerplant. The final fitted distribution was then used to convert each physical trigger and exit threshold into its corresponding cumulative probability, reported as $F(A_{Tr})$ and $F(A_{E})$ in the paper.

**Table B.2.** Estimated parameters of selected distributions

| **Powerplant** | **Selected distribution** | **Parameter 1** | **Estimate** | **SE** | **Parameter 2** | **Estimate** | **SE** | **Selected-distribution BIC** |
| --- | --- | --- | --- | --- | --- | --- | --- | --- |
| Itaipu | Gamma | shape | 132.178 | 13.574 | rate | 0.001238 | 0.000129 | 3,820.134 |
| Belo Monte | Student-t | df | 11.932 | 0.2413 | ncp | 1,351.0111 | 1,931,310 | 715.972 |
| Xingó | Student-t | df | 34.125 | 0.3023 | ncp | 9.832.850 | 280,894 | 3,186.388 |
| Porto Primavera | Student-t | df | 78.589 | 0.7292 | ncp | 5,890.1391 | 1,067,060 | 3,641.380 |

Figures B.1-B.4 present the visual fitting statistics for the chosen distributions.

**Figure B.1.** Affluent Flow – Gamma Fit - Itaipu

**Figure B.2.** Affluent Flow – Student-t Fit – Belo Monte

**Figure B.3.** Affluent Flow – Student-t Fit - Xingó

**Figure B.4.** Average 3-months Precipitation – Weibull Fit – Porto Primavera

It is noteworthy that all best-fitted distributions are heavy-tailed. This is in accordance with the explicatory variables’ histograms (figures B.5 and B.6), which are generally right-skewed.

**Figure B.5.** Histograms of the model’s variables

**Figure B.6.** QQ-Plots of the model’s quantitative variables

With these premises and following the defined pricing method, we computed the PI results for the powerplants, as presented in the paper.
