# Supplementary material for: When There's No November Rain: Developing a Parametric Insurance for Hydroelectric Energy Generators in Brazil
Source: Risk Anal. 2026 Sep 3;46(9):e70347. doi: 10.1111/risa.70347 (PMC13542624; doi:10.1111/risa.70347)
Supplement: Supplementary file 2 [file RISA-46-0-s002.docx]

**Supplementary Appendix IV – Clusters, Probability Distribution Fits and Insurance Results for Water-Storage Powerplants**

**Appendix IV-A. Cluster analyses**

Although we only present the insurance results for run-of-river powerplants in the main paper, we tested what would they be for water-storage plants as well, further showing that the arrangement would not be feasible for this type of powerplant, as already indicated by the copulas models.

As a mirror to the results of run-of-river powerplants, we will present the calculations for the largest water storage energy generator of each region, hence: Salto Santiago (South), Tucuruí (North), Luiz Gonzaga (Northeast) and Ilha Solteira (Southeast). Figure 9 shows the clustering distributions.

Figure A.1 shows the scree plots, which indicate 3 levels are adequate for cluster differentiation.

**Figure A.1.** Scree Plots

Table A.1 summarizes the k-means clustering results. The high Between SS / Total SS values indicate that the three-cluster solution captures a substantial share of the variation in the variables used for clustering.

**Table A.1.** Clustered specifications

| **Powerplant** | **Cluster** | **n** | **Mean generation** | **Mean precipitation** | **Mean useful volume** | **Between SS / Total SS** |
| --- | --- | --- | --- | --- | --- | --- |
| Tucuruí | Low | 88 | 1,799,124 | 589.77 | 53.10 | 87.7% |
|  | Medium | 74 | 3,097,564 | 1,210.23 | 70.99 | 87.7% |
|  | High | 42 | 4,602,235 | 1,724.51 | 87.54 | 87.7% |
| Ilha Solteira | Low | 49 | 788,571 | 699.29 | 32.49 | 87.5% |
|  | Medium | 73 | 1,138,791 | 839.19 | 66.08 | 87.5% |
|  | High | 82 | 1,508,371 | 823.31 | 71.89 | 87.5% |
| Salto Santiago | Low | 55 | 273,26 | 958.72 | 37.82 | 85.7% |
|  | Medium | 71 | 559,275 | 1,216.37 | 62.65 | 85.7% |
|  | High | 78 | 799,58 | 1,303.77 | 87.90 | 85.7% |
| Luiz Gonzaga | Low | 101 | 285,24 | 351.46 | 30.52 | 88.2% |
|  | Medium | 53 | 541,321 | 592.31 | 69.03 | 88.2% |
|  | High | 50 | 735,704 | 480.04 | 78.43 | 88.2% |

Figure A.2 shows the cluster plots for the analyzed water-storage powerplants.

**Figure A.2.** Clusters in a 3-D Perspective

Ilha Solteira presents a great deal of zeros in its volume data, all of each correspond to the severe droughts of 2014 and 2021. Journal news from the time document that the powerplant reservoirs were indeed at the “dead volume” level.^[1]^

**Appendix IV-B. Triggers, Exits and Probability Fits**

From the defined low clusters for each powerplant, we estimated quantiles that could possibly serve as triggers or exits. They are presented in Table B.1.

**Table B.1.** Triggers and Exit Thresholds

|  | Trigger | | | | | Exit Threshold | | |
| --- | --- | --- | --- | --- | --- | --- | --- | --- |
| Powerplant | Average | Median | 35% Quantile | 30% Quantile | 1st Quartile | 5% Quantile | 2.5% Quantile | 1% Quantile |
| Salto Santiago | 37.82 | 35.49 | 30.76 | 28.56 | 24.54 | 12.36 | 9.76 | 8.25 |
| Tucuruí | 53.10 | 41.11 | 29.41 | 28.04 | 26.26 | 19.06 | 16.73 | 15.87 |
| Luiz Gonzaga | 30.52 | 26.15 | 21.52 | 20.47 | 18.88 | 12.28 | 11.30 | 10.56 |
| Ilha Solteira | 32.49 | 22.84 | 0.00 | 0.00 | 0.00 | 0.00 | 0.00 | 0.00 |

Source: own elaboration.

Candidate distributions were fitted separately for each powerplant water-volume series (measured as the percentage of reservoir capacity). The candidate distributions were selected because they represent a range of distributional shapes commonly used in environmental and hydrological applications, including symmetric, skewed, and heavy-tailed alternatives. The objective was not to exhaustively evaluate all possible distributional families, but rather to compare a set of theoretically plausible specifications capable of capturing the distributional characteristics typically observed in reservoir-volume data.

Distribution selection was based on the Bayesian Information Criterion (BIC), which balances model fit and parsimony by penalizing model complexity. Given that the purpose of this stage was to identify a parsimonious distribution for converting physical trigger and exit thresholds into cumulative probabilities, BIC provides an appropriate criterion for model comparison. The selected distribution for each powerplant corresponds to the lowest BIC among the candidate distributions. The best-fit results are presented in Table B.2 and depicted in Figures B.1-B.4.

**Table B.2.** BIC values for candidate distributions

| **Powerplant** | **Exponential BIC** | **Gamma BIC** | **Normal BIC** | **Weibull BIC** | **Student-t BIC** | **Pareto BIC** | **Selected distribution** |
| --- | --- | --- | --- | --- | --- | --- | --- |
| Tucuruí | 2,126.867 | 1,999.016 | 1,977.511 | 1,969.704 | **2,078.317** | 2,148.319 | Weibull |
| Ilha Solteira | 1,903.172 | 1,768.958 | **1,578.144** | 1,608.474 | 781,684,000,000.000 | 1,930.133 | Normal |
| Salto Santiago | 2,120.279 | 1,970.684 | 1,935.526 | 1,932.728 | **2,119.504** | 2,143.494 | Weibull |
| Luiz Gonzaga | 2,027.515 | 1,936.219 | 1,957.343 | 1,929.941 | **1,996.058** | 2,044.516 | Weibull |

Table B.3 reports the estimated parameters of the selected distribution for each powerplant. The final fitted distribution was then used to convert each physical trigger and exit threshold into its corresponding cumulative probability, reported as $F(V_{Tr})$ and $F(V_{E})$ in the paper.

**Table B.3.** Estimated parameters of selected distributions

| **Powerplant** | **Selected distribution** | **Parameter 1** | **Estimate** | **SE** | **Parameter 2** | **Estimate** | **SE** | **Selected-distribution BIC** |
| --- | --- | --- | --- | --- | --- | --- | --- | --- |
| Tucuruí | Weibull | shape | 24.479 | 0.1470 | scale | 753585 | 22595 | 1,969.704 |
| Ilha Solteira | Normal | mean | 603.453 | 18889 | sd | 269.796 | 13.357 | 1,933.956 |
| Salto Santiago | Weibull | shape | 26.848 | 0.1587 | scale | 738.350 | 20.160 | 1,932.728 |
| Luiz Gonzaga | Weibull | shape | 19.314 | 0.1095 | scale | 591.370 | 22.608 | 1,929.941 |

For Ilha Solteira, the Weibull fitting procedure initially required filtering strictly positive useful-volume observations because of the presence of zero values. However, the Normal distribution produced the lowest BIC among the candidate distributions and was therefore selected. The Normal distribution was then refitted using the full sample, including zero values, and its fitted CDF was used to compute the cumulative probabilities associated with the trigger and exit thresholds.

Figures B.1-B.4 present the visual fitting statistics for the chosen distributions.

**Figure B.1.** Useful Volume – Weibull Fit – Salto Santiago

**Figure B.2.** Useful Volume – Weibull Fit - Tucuruí

**Figure B.3.** Useful Volume – Weibull Fit – Luiz Gonzaga

**Figure B.4.** Useful Volume – Normal Fit – Ilha Solteira

The selected distributions reflect the heterogeneous distributional characteristics of reservoir-volume data. While most series were better represented by Weibull distributions, Ilha Solteira was best fitted by a Normal distribution, likely reflecting the influence of reservoir-management practices and the presence of extreme drought episodes documented in the sample. This is in accordance with the explicatory variables’ histograms (figures B.5 and B.6), which are generally right-skewed.

**Figure B.5.** Histograms of the model’s variables

**Figure B.6.** QQ-Plots of the model’s quantitative variables

Table B.4 presents the results of determine $F\left( V_{Tr} \right)$ and $F\left( V_{E} \right)$ for each possible threshold.

**Table B.4.** Distributions and Frequency Probabilities

| $\boldsymbol{F}\left( \boldsymbol{V}_{\boldsymbol{Tr}} \right)$ | | | | | | | $\boldsymbol{F}\left( \boldsymbol{V}_{\boldsymbol{E}} \right)$ | |  |
| --- | --- | --- | --- | --- | --- | --- | --- | --- | --- |
| Powerplant | Distribution of V | Average | Median | 35% Quantile | 30% Quantile | 1st Quartile | 5% Quantile | 2.5% Quantile | 1% Quantile |
| Salto Santiago | Weibull | 0.1529 | 0.1306 | 0.0909 | 0.0751 | 0.0506 | 0.0082 | 0.0044 | 0.0028 |
| Tucuruí | Weibull | 0.3459 | 0.2030 | 0.0951 | 0.0851 | 0.0729 | 0.0340 | 0.0248 | 0.0218 |
| Luiz Gonzaga | Weibull | 0.2432 | 0.1868 | 0.1323 | 0.1209 | 0.1044 | 0.0469 | 0.0401 | 0.0352 |
| Ilha Solteira | Normal | 0.1509 | 0.0822 | 0.0127 | 0.0127 | 0.0127 | 0.0127 | 0.0127 | 0.0127 |

Source: own elaboration.

With these premises and following the defined pricing method, we computed the PI results for the powerplants.

**Appendix IV-C. Insurance Results**

Just as in the run-of-river powerplants exercise, for the water storage powerplants the triggers were chosen in order to standardize the claims frequency level around 4%, and as the exit threshold choice did not show expressive variations on the payout or premium amounts, we kept the intermediate level (2.5) as exit for all powerplants. Results are presented in Table C.1

**Table C.1.** Insurance Design Results

| Powerplant | Chosen Triggers (Quantiles) | Chosen Exit thresholds (Quantiles) | Expected Claims Frequency ($\mathbb{P}\left( I_{i} \right)$) | Observed Claims Frequency | Annual Payout (BRL) | Annual Risk Premium (BRL) | Payouts during droughts of 2014-2016 or 2021-2022? |
| --- | --- | --- | --- | --- | --- | --- | --- |
| Salto Santiago | 25% Quantile | 2.5% Quantile | 4.6% | 6.9% | 2,090,688,043 | 96,589,788 | Yes |
| Tucuruí | 25% Quantile | 2.5% Quantile | 4.8% | 11.8% | 2,397,102,886 | 115,300,649 | Yes |
| Luiz Gonzaga | 25% Quantile | 2.5% Quantile | 6.4% | 12.7% | 1,912,873,779 | 122,997,784 | Yes |
| Ilha Solteira | 50% Quantile | 2.5% Quantile | 7.0% | 12.7% | 11,093,412,293 | 770,992,154 | Yes |

Table C.1 shows that, although the same pricing procedure can technically be applied to water-storage powerplants, the resulting insurance design is less suitable than for run-of-river plants. The observed claims frequencies are systematically higher than the expected frequencies implied by the selected triggers, especially for Tucuruí, Luiz Gonzaga, and Ilha Solteira. While the selected thresholds were chosen to keep the expected claims frequency around the 4–7% range, the observed claims frequency reaches 11.8% for Tucuruí and 12.7% for both Luiz Gonzaga and Ilha Solteira. This indicates that the trigger structure does not reproduce historical claim occurrence as closely as desired for these plants.

The resulting payouts and premiums also point to weaker actuarial feasibility for water-storage plants under this same product structure. In these cases, the contract would require very large payouts and premiums without a sufficiently stable correspondence between the selected trigger and the insured generation shortfall. Ilha Solteira is the clearest example of this limitation, as its useful-volume series includes several zero observations associated with severe drought episodes, which creates a more irregular risk profile and makes the distribution-fitting step more sensitive. Therefore, the issue is not merely that the estimated amounts are high, but that the trigger does not appear to provide a sufficiently reliable and insurable representation of the underlying generation risk.

Overall, this supplementary exercise supports the decision not to include water-storage plants in the main proposed product design. This result is consistent with the vine copula analysis reported in the main paper and in the Supplementary Appendix II, which indicates a more diffuse dependence structure for water-storage powerplants than for run-of-river plants. While the run-of-river cases show a clearer causal pathway from climatic variables to affluent flow and then to electricity generation, the water-storage cases present weaker and less direct links between reservoir volume, hydrometeorological conditions, and generation. This reflects the operational nature of storage plants, whose output also depends on reservoir management decisions, storage capacity, dispatch rules, and intertemporal water-use strategies. Consequently, applying the same parametric structure to water-storage plants would likely generate higher basis risk and potential moral hazard concerns, reinforcing that a dedicated product design would be required for this segment.

1. 2021: <https://oglobo.globo.com/economia/reservatorios-de-cinco-usinas-no-sudeste-tem-volume-menor-que-10-veja-estrago-da-crise-hidrica-na-regiao-25199378>; <https://g1.globo.com/jornal-nacional/noticia/2021/09/18/maior-reservatorio-de-sp-chega-a-nivel-critico-e-interrompe-navegacao-numa-das-principais-hidrovias-do-pais.ghtml>; <https://www.metropoles.com/brasil/maior-hidreletrica-de-sp-atinge-0-do-reservatorio-e-chega-ao-volume-morto>; 2014: <https://www.estadao.com.br/economia/reservatorio-de-ilha-solteira-e-tres-irmaos-chega-a-zero-imp-/>; <https://www.campograndenews.com.br/economia/hidreletrica-de-ms-tem-a-pior-situacao-do-pais-reservatorio-esta-zerado>; <https://agenciabrasil.ebc.com.br/geral/noticia/2014-10/nivel-dagua-dos-principais-reservatorios-do-pais-e-inferior-aos-indices-de>
