# Supplementary material for: When There's No November Rain: Developing a Parametric Insurance for Hydroelectric Energy Generators in Brazil
Source: Risk Anal. 2026 Sep 3;46(9):e70347. doi: 10.1111/risa.70347 (PMC13542624; doi:10.1111/risa.70347)
Supplement: Supplementary file 3 [file RISA-46-0-s003.docx]

**Supplementary Appendix II: Complementary Details on the Vine Copula Estimation and Analyses**

**Appendix II-A. ARIMA-GARCH specifications for each powerplant**

**Table A.1.** ARIMA-GARCH specifications for each powerplant

| **Powerplant** | **Variable** | **ARIMA(p,d,q) specification** | **GARCH(m,n) specification** | **Ljung-Box  p-value** | **LM-Arch  p-value** |
| --- | --- | --- | --- | --- | --- |
| Itaipú | Energy | (6,1,0) | - | 0.1376 | 0.9830 |
|  | Affluent flow | (1,1,1) | (1,2) | 0.1029 | 0.1302 |
|  | Precipitation | (12,1,1) | - | 0.4414 | 0.2493 |
|  | Temperature | (9,1,1) | (2,1) | 0.1551 | 0.8686 |
|  | Wind speed | (0,1,5) | (1,1) | 0.0512 | 0.3799 |
|  | Humidity | (6,1,1) | - | 0.0535 | 0.1749 |
| Belo Monte | Energy | (3,1,0) | - | 0.0639 | 0.2670 |
|  | Affluent flow | (0,1,2) | - | 0.1367 | 0.3805 |
|  | Precipitation | (3,1,0) | - | 0.1816 | 0.7290 |
|  | Temperature | (5,1,0) | - | 0.4989 | 0.1119 |
|  | Wind speed | (0,1,1) | - | 0.3594 | 0.8641 |
|  | Humidity | (4,1,0) | - | 0.1046 | 0.8294 |
| Xingó | Energy | (6,1,0) | - | 0.0949 | 0.0801 |
|  | Affluent flow | (1,1,3) | (1,2) | 0.9889 | 0.9999 |
|  | Precipitation | (6,1,3) | - | 0.3880 | 0.9999 |
|  | Temperature | (12,1,3) | - | 0.9995 | 0.7888 |
|  | Wind speed | (6,1,4) | - | 0.2831 | 0.4081 |
|  | Humidity | (2,1,2) | - | 0.1039 | 0.1858 |
| Porto Primavera | Energy | (12,1,3) | (1,2) | 0.7660 | 0.0738 |
|  | Affluent flow | (8,1,2) | (1,1) | 0.2146 | 0.1796 |
|  | Precipitation | (12,1,2) | - | 0.6389 | 0.1301 |
|  | Temperature | (12,1,0) | - | 0.3353 | 0.0629 |
|  | Wind speed | (0,1,5) | - | 0.6070 | 0.1739 |
|  | Humidity | (6,1,3) | - | 0.3556 | 0.1020 |
| **Powerplant** | **Variable** | **ARIMA(p,d,q) specification** | **GARCH(m,n) specification** | **Ljung-Box  p-value** | **LM-Arch  p-value** |
| Tucuruí | Energy | (12,1,0) | - | 0.8297 | 0.0652 |
|  | Water volume | (4,1,3) | (1,1) | 0.2644 | 0.0783 |
|  | Precipitation | (6,1,4) | (1,2) | 0.1443 | 0.1527 |
|  | Temperature | (9,1,3) | - | 0.5154 | 0.1352 |
|  | Wind speed | (4,1,1) | (1,1) | 0.4914 | 0.0735 |
|  | Humidity | (4,1,1) | (1,1) | 0.3850 | 0.3368 |
| Ilha Solteira | Energy | (12,1,12) | - | 0.9469 | 0.2661 |
|  | Water volume | (5,1,2) | - | 0.8102 | 0.9421 |
|  | Precipitation | (6,1,5) | (1,1) | 0.1592 | 0.0937 |
|  | Temperature | (7,1,2) | - | 0.2509 | 0.7753 |
|  | Wind speed | (4,1,3) | - | 0.5092 | 0.0505 |
|  | Humidity | (6,1,3) | - | 0.0568 | 0.7145 |
| Salto Santiago | Energy | (1,1,2) | - | 0.8245 | 0.8675 |
|  | Water volume | (3,1,2) | - | 0.5630 | 0.8884 |
|  | Precipitation | (4,1,4) | - | 0.7397 | 0.2524 |
|  | Temperature | (9,1,1) | (1,1) | 0.1058 | 0.7292 |
|  | Wind speed | (4,1,2) | - | 0.1611 | 0.4471 |
|  | Humidity | (6,1,1) | - | 0.1009 | 0.5622 |
| Luiz Gonzaga | Energy | (1,1,1) | - | 0.1153 | 0.0700 |
|  | Water volume | (6,1,2) | - | 0.2615 | 0.4064 |
|  | Precipitation | (9,1,2) | - | 0.3547 | 0.9981 |
|  | Temperature | (9,1,2) | - | 0.6911 | 0.4406 |
|  | Wind speed | (6,1,1) | - | 0.1862 | 0.7669 |
|  | Humidity | (2,1,2) | - | 0.6409 | 0.8070 |

**Appendix II-B.** Vine copulas for run-of-river powerplants

**Figure B.1.** Network representing Xingó’s Vine Copula

**Figure B.2.** Network representing Porto Primavera’s Vine Copula

**Figure B.3.** Network representing Belo Monte’s Vine Copula

**Appendix II-C.** Vine copulas for water-storage powerplants

**Figure C.1.** Network representing Luiz Gonzaga’s Vine Copula

**Figure C.2.** Network representing Salto Santiago’s Vine Copula
