# Supplementary material for: When There's No November Rain: Developing a Parametric Insurance for Hydroelectric Energy Generators in Brazil
Source: Risk Anal. 2026 Sep 3;46(9):e70347. doi: 10.1111/risa.70347 (PMC13542624; doi:10.1111/risa.70347)
Supplement: Supplementary file 4 [file RISA-46-0-s004.docx]

**Supplementary Appendix I: Spatial Analysis Framework and Estimation Details**

# Appendix I-A. Spatial analysis framework

This appendix provides a detailed description of the spatial procedures adopted in the empirical analysis. It documents the construction of the hydrological spatial matrix, the treatment of the unbalanced panel, the diagnostic procedures for residual spatial dependence (Newey and West 1987; Andrews 1991; Anselin et al. 1996), the IV-GMM-HAC estimation of the SAR specification (Kelejian and Prucha 1998; 1999), the robustness exercises, and the calculation of spatial impacts (LeSage and Pace 2011). The goal is to make the empirical strategy transparent and reproducible.

# A.1. Data preparation and panel construction

The starting point of the analysis is a monthly panel of hydroelectric plants containing electricity generation and hydrometeorological covariates. Let $i=1,\ldots,n_{t}$index plants active in month $t=1,\ldots,T$, where $n_{t}$may vary over time. The working panel is therefore unbalanced.

## A.1.1. Time standardization

The monthly time index is standardized to the first day of each month, so that all observations are mapped to a common monthly key. This ensures consistent alignment between the plant-level data and the monthly hydrological network.

## A.1.2. Post-entry unbalanced panel

For each plant, the entry month is defined as the first month in which electricity generation is strictly positive. Let $t_{i}^{0}$denote this plant-specific entry date. The analysis is then conducted on the observed post-entry panel:

$$\mathcal{T}_{i}=\{t:t\geq t_{i}^{0}\}.$$

This construction avoids imposing an artificial balanced panel that would either discard relevant observations or attribute a spatial structure to plants not yet operating in a given month.

## A.1.3. Imputation rules for selected variables

For post-entry months that are present in the plant-month support but missing in the original data, only a limited set of variables is imputed with zero, namely electricity generation, useful reservoir volume, and affluent flow. The rationale is that these are operational variables for which a zero value is meaningful in the completed post-entry support. Other variables remain as originally observed.

## A.1.4. Centering and interaction terms

The main hydrometeorological variables are centered using the mean computed from the original observed data, before post-entry completion. Let $x_{it}$denote any of the original variables and $\bar{x}$its reference mean. The centered variable is:

$$x_{it}^{c}=x_{it}-\bar{x}.$$

The following centered covariates are used in the baseline specification:

- $\text{volume\_util}_{\boldsymbol{it}}^{\boldsymbol{c}}$
- $\text{vazao\_afluente}_{\boldsymbol{it}}^{\boldsymbol{c}}$
- $\text{precip\_media\_3m}_{\boldsymbol{it}}^{\boldsymbol{c}}$
- $\text{vento}_{\boldsymbol{it}}^{\boldsymbol{c}}$
- $\text{umidade}_{\boldsymbol{it}}^{\boldsymbol{c}}$

From these, three interaction terms are defined:

$$UV\_x\_P_{it}=\text{volume\_util}_{it}^{c}\times\text{precip\_media\_3m}_{it}^{c},$$

$$VA\_x\_P_{it}=\text{vazao\_afluente}_{it}^{c}\times\text{precip\_media\_3m}_{it}^{c},$$

$$W\_x\_H_{it}=\text{vento}_{it}^{c}\times\text{umidade}_{it}^{c}.$$

Construction of the hydrological spatial matrix

## A.1.5. Motivation

The relevant notion of proximity in this application is hydrological, not Euclidean. Plants connected along the same river network may be strongly dependent even when they are geographically distant, whereas geographically close plants in different basins may have weak hydrological interaction. For that reason, the spatial matrix is built from the hydrological network rather than from geographic distance.

## A.1.6. Monthly hydrological adjacency

For each month $t$, let $W_{t}$be the $n_{t}\times n_{t}$ weighting matrix defined over the active plants in that month. Each element $w_{ij,t}$is binary and directional:

$$w_{ij,t}=\left\{ \begin{matrix} 1, & \text{if plant }j\text{ is a first-order upstream hydrological neighbor of plant }i\text{ in month }t, \\ 0, & \text{otherwise.} \end{matrix} \right.$$

Rows correspond to downstream plants and columns to upstream plants. Hence, a nonzero entry means that plant $j$can affect plant $i$through the river network. The matrix is restricted to first-order hydrological links and the diagonal is set equal to zero:

$$w_{ii,t}=0.$$

## A.1.7. Boundedness

Because the matrix is binary, directional, and restricted to first-order hydrological adjacency, the absolute row sums are uniformly bounded. Under the maintained hydrological network structure, column sums also remain bounded. Therefore, the sequence $\left\{ W_{t}{\}}_{t=1}^{T} \right.$satisfies the standard boundedness conditions commonly imposed in spatial asymptotics.

## A.1.8. Time variation

Since the set of active plants varies over time, the spatial matrix also varies with time. The empirical analysis therefore uses a sequence of monthly matrices $W_{t}$, rather than a single time-invariant $W$.

**Appendix I-B. Spatial Diagnostics and Estimation in an Unbalanced Panel with Time-Varying Hydrological Matrices**

# B.1. Setup, notation, and scope

This appendix presents the econometric details of the spatial analysis used in the article. The emphasis is on the technical adaptation of residual Moran diagnostics, LM specification tests, and IV-GMM-HAC estimation to an unbalanced monthly panel with period-specific hydrological weighting matrices.

Let $t = 1, \ldots, T$ index months and let $n_{t}$ denote the number of active hydroelectric plants in period $t$. The panel is unbalanced, so *n_t_* is allowed to vary over time. For each month $t$, define:

- $\boldsymbol{E}_{\boldsymbol{t}}$: $\boldsymbol{n}_{\boldsymbol{t}}\boldsymbol{\times1}$ vector of electricity generation,
- $\boldsymbol{X}_{\boldsymbol{t}}$: $\boldsymbol{n}_{\boldsymbol{t}}\boldsymbol{\times k}$ matrix of exogenous covariates,
- $\boldsymbol{W}_{\boldsymbol{t}}$: $\boldsymbol{n}_{\boldsymbol{t}}\boldsymbol{\times}\boldsymbol{n}_{\boldsymbol{t}}$ hydrological weighting matrix.

The preferred empirical specification is a spatial autoregressive model with plant and time fixed effects:

$$E_{t}=\rho W_{t}E_{t}+X_{t}\beta+\mu_{i}+\tau_{t}+u_{t}, t=1,\ldots, T$$

The matrices $W_{t}$ are binary, directional, and based on first-order hydrological adjacency. Rows correspond to downstream plants and columns to upstream plants. Diagonal elements are set equal to zero.

For notational convenience, define the stacked vectors

$$E=\left( E_{1}^{'},\ldots,E_{T}^{'} \right)^{'}, e=\left( e_{1}^{'},\ldots,e_{T}^{'} \right)^{'}$$

and the block-diagonal matrix

$$W=\mathrm{diag}\left( W_{1},\ldots,W_{T} \right)$$

Although the panel is unbalanced, this representation is well defined because each block $W_{t}$ is conformable with the active plant set in period $t$.

In what follows, $n_{t}$ denotes the number of active plants in period $t$. The total number of stacked observations is $N=\sum_{t=1}^{T} n_{t}$, and $S_{0}$ denotes the total sum of weights in the stacked matrix. The symbol $M_{Z}$ denotes the residual-maker associated with the relevant null model after partialling out the regressors and the fixed effects. The vector $g_{t}\left( \theta\right)$ denotes the period-specific moment condition used in the GMM procedure, and $\hat{S}_{HAC}$ denotes the HAC estimator of the long-run covariance matrix of the resulting moment sequence.

# B.2. Regularity conditions

The results below rely on the following assumptions.

## Assumption A1 (Bounded spatial matrices)

For all $t$, the matrices $W_{t}$ have zero diagonal and uniformly bounded row and column sums. That is, there exists a constant $C<\infty$ such that

$$\sup_{t} \max_{i} \sum_{j} \left| w_{ij,t} \right|\leq C, \sup_{t} \max_{j} \sum_{j} \left| w_{ij,t} \right|\leq C$$

## Assumption A2 (Moment conditions)

The regressors and disturbances have finite second moments, and the fourth moments required for the asymptotic approximation of quadratic forms also exist.

## Assumption A3 (Weak temporal dependence)

The process $\left\{ g_{t}\left( \theta_{0} \right) \right\}^{T}$ is $\alpha$-mixing with mixing coefficients $\alpha\left( m \right)$ satisfying

$$\sum_{m=1}^{\infty} \alpha\left( m \right)^{1-\frac{2}{r}}$$

for some *r* > 2. This condition is sufficient for consistency of the Bartlett-kernel HAC estimator of the long-run covariance matrix.

## Assumption A4 (Identification)

After removing plant and time fixed effects, the instrument matrix has full column rank and contains at least as many columns as the number of structural regressors.

A substantive justification for the preferred instrument set is the following. The terms $W_{t}X_{t}$ capture first-order hydrological neighbors’ exogenous conditions, while $W_{t}^{2}X_{t}$ captures second-order hydrological exposure. These variables are excluded from the structural equation but affect the spatial lag of generation through the hydrological network, which provides the exclusion logic underlying the IV construction.

t

## Assumption A5 (Non-singularity)

The true parameter $\rho$ satisfies

$$\left| \rho\right|<\frac{1}{\lambda_{\max}\left( W_{t} \right)}, for all t,$$

where $\lambda_{\max}\left( W_{t} \right)$ denotes the spectral radius of $W_{t}$. This ensures that $\left( I - \rho W_{t} \right)$ is invertible for all $t$ and that the Neumann-series expansion of $\left( I - \rho W_{t} \right)^{-1}$ converges absolutely.

# B.3. Residual spatial diagnostics in the unbalanced panel

## B.3.1. Global Moran's *I* on the stacked panel

To evaluate residual spatial autocorrelation, we compute a global Moran statistic on the stacked residual vector:

$$I=\frac{N}{S_{0}}\frac{e^{'}We}{e^{'}e},$$

where

$$S_{0}=\iota_{n_{t}}^{'}W_{t}\iota_{n_{t}}=\sum_{t=1}^{T} \sum_{i=1}^{n_{t}} \sum_{j=1}^{n_{t}} w_{ij,t}.$$

## Proposition A1 (Stacked representation of Moran's *I*)

Under Assumption A1, the global panel Moran statistic is well defined and equals the ratio of the sum of period-specific quadratic forms to the sum of period-specific residual sums of squares.

Proof

Since $W$ is block-diagonal, all off-diagonal cross-period blocks are zero. Therefore,

$$e^{'}We=\sum_{t=1}^{T} e_{t}^{'}W_{t}e_{t}.$$

Likewise, because $e$ is obtained by stacking the residual vectors,

$$e^{'}e=\sum_{t=1}^{T} e_{t}^{'}e_{t}.$$

Assumption A1 guarantees boundedness of the blocks, so the numerator is finite whenever residuals have finite second moments. The denominator is finite and positive except in degenerate cases. It follows that *I* is well defined.

## B.3.2. Asymptotic approximation

Inference for the stacked Moran statistic is based on the standard approximation

$$\mathbb{E}\left[ I \right]=-\frac{1}{N-1},$$

with variance determined by the stacked quantities *S*_0_, *S*_1_, and *S*_2_, where

$$S_{1}=\frac{1}{2}\sum_{i} \sum_{j} \left( w_{ij}+w_{ji} \right)^{2}, S_{2}=\sum_{i} \left( w_{i+}+w_{+i} \right)^{2}, w_{i+}=\sum_{j} w_{ij}, w_{+i}=\sum_{j} w_{ji}.$$

Because the stacked matrix is block-diagonal, both *S*_1_ and *S*_2_ are additive across periods. The standardized statistic is therefore

$$Z_{I}=\frac{I-\mathbb{E}\left[ I \right]}{\sqrt{\mathrm{Var}\left( I \right)}},$$

## B.3.3. LM diagnostics under the OLS null

Let $e_{t}$ denote the residuals from the two-way fixed-effects OLS null, and define

$$Wy_{t}=W_{t}E_{t}, We_{t}=W_{t}e_{t},$$

The period-aggregated lag and error scores are

$$q_{\rho}=\frac{\sum_{t=1}^{T} e_{t}^{'}W_{t}E_{t}}{\hat{\sigma}^{2}}, q_{\lambda}=\frac{\sum_{t=1}^{T} e_{t}^{'}W_{t}e_{t}}{\hat{\sigma}^{2}}$$

Let $Z$ denote the matrix of within-transformed regressors under the OLS null, that is, the exogenous variables after removing plant and time fixed effects. Define $M_{Z}=I_{N}-Z\left( Z^{'}Z \right)^{-1}Z^{'},$ where $I_{N}$ is the identity matrix of conformable dimension. Then $M_{Z}$is the residual-maker associated with the null model. If $Wy$ denotes the stacked vector of spatial lags of the dependent variable, define

$$D=\frac{\left( Wy \right)^{'}M_{Z}\left( Wy \right)}{\hat{\sigma}^{2}}+J.$$

where

$$J=\sum_{t=1}^{T} \mathrm{tr}\left( W_{t}^{'}W_{t}+W_{t}^{2} \right)$$

The simple LM statistics are then

$$LM_{lag}=\frac{q_{\rho}^{2}}{D}, LM_{err}=\frac{q_{\lambda}^{2}}{J}.$$

## Proposition A2 (Block-diagonal decomposition of the LM statistics)

Under Assumptions A1–A4, the LM scores and their trace-based information terms decompose additively across periods.

Proof

All quantities entering the LM statistics depend on bilinear or quadratic forms involving $W$, the dependent variable, and the residuals. Since $W$ is block-diagonal, all cross-period interaction terms vanish. Therefore,

$$e^{'}WE=\sum_{t=1}^{T} e_{t}^{'}W_{t}E_{t}, e^{'}We=\sum_{t=1}^{T} e_{t}^{'}W_{t}e_{t}.$$

Similarly, the trace term is additive because

$$\mathrm{tr}\left( W^{'}W+W^{2} \right)=\sum_{t=1}^{T} \mathrm{tr}\left( W_{t}^{'}W_{t}+W_{t}^{2} \right).$$

Hence the LM statistics are sums of period-specific score contributions and period-specific trace terms. The unbalanced nature of the panel changes only the dimensions of the blocks and does not alter the algebraic decomposition. $\square$

## B.3.4. Decomposed lag diagnostics for asymmetric matrices

When the interaction matrix is asymmetric, the usual quadratic residual diagnostics are not informative about directional relevance. In particular, Chagas (2026) shows that statistics based on quadratic residual forms, such as Moran’s $I$and the LM-error statistic, are exactly invariant to the antisymmetric component of the matrix. By contrast, lag-type bilinear diagnostics retain directional information. This distinction is especially relevant in hydrological networks, where direction is part of the substantive propagation mechanism.

To address this issue, write the period-specific matrix as

$$W_{t}=W_{s,t}+W_{a,t},W_{s,t}=\frac{W_{t}+W_{t}^{'}}{2},W_{a,t}=\frac{W_{t}-W_{t}^{'}}{2},$$

where $W_{s,t}$is the symmetric component and $W_{a,t}$is the antisymmetric component. Under the restricted model, define the decomposed score contributions

$$q_{s,t}=\frac{e_{t}^{'}W_{s,t}Y_{t}-\hat{\sigma}^{2}\mathrm{tr}(M_{t}W_{s,t}M_{t})}{\hat{\sigma}^{2}},q_{a,t}=\frac{e_{t}^{'}W_{a,t}Y_{t}}{\hat{\sigma}^{2}},$$

and aggregate them over time as

$$q_{s}=\sum_{t=1}^{T} q_{s,t},q_{a}=\sum_{t=1}^{T} q_{a,t},q=(q_{s},q_{a})^{'}.$$

Let $\hat{I}_{N}$denote the outer-product estimator of the covariance matrix of $q$. Following Chagas (2026), three decomposed lag tests are then considered. The joint statistic

$$LM_{s,a}=q^{'}\hat{I}_{N}^{-1}q$$

tests whether network propagation is present through either channel. The conditional directional statistic

$$LM_{a\mid s}=\frac{\left( q_{a} - \hat{I}_{as}\hat{I}_{ss}^{-1}q_{s} \right)^{2}}{\hat{I}_{aa}-\hat{I}_{as}\hat{I}_{ss}^{-1}\hat{I}_{sa}}$$

tests whether the antisymmetric component contributes information beyond symmetric exposure. The corresponding contextual statistic

$$LM_{s\mid a}=\frac{\left( q_{s} - \hat{I}_{sa}\hat{I}_{aa}^{-1}q_{a} \right)^{2}}{\hat{I}_{ss}-\hat{I}_{sa}\hat{I}_{aa}^{-1}\hat{I}_{as}}$$

tests whether the symmetric component remains relevant after conditioning on the directional one. In the present application, $LM_{a\mid s}$is particularly informative because it asks whether upstream–downstream orientation matters beyond simple hydrological connectivity.

## B.3.5. LM diagnostics under the SLX null

The same logic is applied under the SLX null, except that the residuals and the projection matrix are now those from the fixed-effects SLX specification with regressors $[X_{t}, W_{t}X_{t}]$. The resulting LM and robust LM statistics therefore test whether additional lag or error dependence remains after conditioning on spatially lagged covariates.

# B.4. IV-GMM-HAC estimation of the SAR model

## B.4.1. Motivation

The preferred model is estimated by instrumental variables generalized method of moments (IV-GMM), following the logic of Kelejian and Prucha's spatial GMM-IV approach, adapted here to an unbalanced panel with time-varying hydrological matrices. This choice is motivated by three features of the data: (i) the panel is unbalanced, (ii) the spatial matrix varies over time, and (iii) the residual process displays heteroskedasticity and temporal dependence. Under these conditions, balanced-panel likelihood procedures based on a fixed spatial matrix are not appropriate.

## B.4.2. Fixed-effects removal

Plant and time fixed effects are removed before estimation by residualizing each variable with respect to the two-way fixed-effects structure. Let *E_t_*, *X_t_*, and *W_t_E_t_* denote the corresponding within-transformed objects. The transformed structural equation is

$$\tilde{E}_{t}=\rho\tilde{W_{t}E_{t}}+\tilde{X}_{t}\beta+\tilde{u}_{t}$$

Because the transformed model includes a spatially lagged dependent variable, the within-transformation may in principle induce an incidental-parameter bias when $T$ is small. In the present application, however, the monthly panel is sufficiently long for such bias to be of order $O(1/T )$ and therefore expected to be limited. The robustness analysis further serves as a practical check on the stability of the estimated spatial parameter.

## B.4.3. Instrument set

The endogenous regressor is the spatial lag of the dependent variable. The preferred baseline instrument set is

$$Z_{t}=\left[ X_{t}, W_{t}X_{t},W_{t}^{2}X_{t} \right]$$

The first block contains the exogenous variables, the second contains first-order hydrological spatial lags, and the third contains second-order spatial lags. The terms $W_{t}^{2}X_{t}$ represent exogenous conditions at second-order hydrological neighbors. Their identifying role is that they affect current generation only through the spatial lag of generation and not directly through the structural equation, thereby supplying the exclusion logic of the IV strategy.

t

## B.4.4. Period-specific moments

For each month $t$, let $u_{t}(\theta)$ denote the structural residual implied by parameter vector $\theta$. The estimation uses the period-specific moment vector

$$g_{t}\left( \theta\right)=\frac{1}{\sqrt{N}}Z_{t}^{'}u_{t}\left( \theta\right), N=\sum_{t=1}^{T} n_{t}$$

This normalization puts all monthly moments on a common scale compatible with the asymptotic variance of the stacked sample moments.

## B.4.5. HAC weighting matrix

Because the sequence $\left\{ g_{t} \right\}_{t=1}^{T}$ may display temporal dependence, the GMM weighting matrix is based on a HAC estimator of the long-run covariance matrix of the period-specific moments. Specifically,

$$\hat{S}_{HAC}=\hat{\Gamma}_{0}+\sum_{\mathcal{l=}1}^{L} w_{\mathcal{l}}\left( \hat{\Gamma}_{\mathcal{l}}+\hat{\Gamma}_{\mathcal{l}}^{'} \right),$$

where

$$\hat{\Gamma}_{\mathcal{l}}=\frac{1}{T}\sum_{t\mathcal{=l+}1}^{T} g_{t}\left( \hat{\theta} \right)g_{t\mathcal{-l}}\left( \hat{\theta} \right)^{'}\mathcal{, l=}0,1,\ldots,L$$

and $w_{\mathcal{l}} = 1 - \mathcal{l/(}L + 1)$ are Bartlett weights. In the baseline specification, the bandwidth follows the Newey–West rule

$$L=\left[ 4\left( \frac{T}{100} \right)^{2/9} \right],$$

and is then perturbed upward and downward in the robustness analysis.

## Proposition A4 (Consistency of the block-HAC estimator)

Under Assumptions A1–A3, and if the bandwidth satisfies *L* → ∞ and *L*/*T* → 0 as *T* → ∞, the estimator $\hat{S}_{HAC}$ consistently estimates the long-run covariance matrix of the sample moments,

$$S=\Gamma_{0}+\sum_{\mathcal{l=}1}^{\infty} \left( \Gamma_{\mathcal{l}}+\Gamma_{\mathcal{l}}^{'} \right), \Gamma_{\mathcal{l}}\mathbb{=E[}g_{t}\left( \theta_{0} \right)g_{t\mathcal{-l}}(\theta_{0})' ]$$

Proof

Let $\theta_{0}$ denote the true parameter vector and write $g_{t}=g_{t}(\theta_{0})$. The object of interest is the long-run covariance matrix of the time-indexed sequence $\left\{ g_{t} \right\}_{t=1}^{T}$. Since the moments are indexed by time, the relevant dependence for HAC estimation is temporal.

*t*=1

Each $g_{t}$ is constructed from period-$t$ quantities only, namely $W_{t}$, the corresponding instrument matrix, and the structural residual vector. Because the spatial matrix is block-specific, all contemporaneous spatial dependence is internal to period $t$. There are no cross-period spatial interaction terms in the definition of $g_{t}$. The role of the HAC estimator is therefore to account for serial dependence in the sequence $\{g_{t}\}$.

Assumption A1 guarantees that the matrices $W_{t}$ have uniformly bounded row and column sums. Combined with Assumption A2, this implies that the linear and bilinear forms entering $g_{t}$ have uniformly bounded second moments. Hence,

$${\sup_{t} \mathbb{E}\left\| g_{t} \right\|}^{2}<\infty$$

so the covariance matrices $\Gamma_{\mathcal{l}}$ are well defined. Assumption A3 implies that $\{g_{t}\}$ is weakly dependent over time in a form strong enough to ensure the convergence of sample autocovariances. Therefore, for each fixed lag $\mathcal{l}$,

$$\hat{\Gamma}_{\mathcal{l}}\underset{\to}{p}\Gamma\mathcal{\_l}$$

The bandwidth conditions $L \to\infty$ and $L/T \to0$ ensure that truncation bias vanishes while estimation noise from high-order sample autocovariances becomes asymptotically negligible. It follows that

$$\hat{S}_{HAC}\underset{\to}{p}S.$$

Finally, the fact that the panel is unbalanced does not affect this conclusion. The varying cross-sectional dimension $n_{t}$ influences only the internal composition of each period-specific moment, not the time-series structure of the sequence $\{g_{t}\}$. Since the period-specific moments remain uniformly well behaved under Assumptions A1 and A2, the standard HAC argument applies to the aggregated spatial moment process. $\square$

## B.4.6. Two-step estimation and overidentification test

The estimator is implemented in two steps. In the first step, a preliminary weighting matrix is obtained from the covariance matrix of the instruments. In the second step, residuals from the first-stage estimate are used to construct $\hat{S}_{HAC}$, whose inverse defines the final GMM weighting matrix.

The overidentification test is based on the standard GMM statistic

$$J=N\bar{g}\left( \hat{\theta} \right)^{'}\hat{A}\bar{g}\left( \hat{\theta} \right),$$

where $\bar{g}(\hat{\theta})$ is the sample average of the stacked moments and $\hat{A}=\hat{S}_{HAC}^{-1}$. Under correct specification and valid instruments, $J$ is asymptotically chi-square with degrees of freedom equal to the number of overidentifying restrictions.

# B.5. Robustness analysis

The robustness analysis varies two ingredients:

1. the instrument set,
2. the HAC bandwidth.

The estimated spatial autoregressive coefficient remains positive and significant across admissible combinations. This is important because it shows that the estimated propagation effect is not an artifact of a particular tuning choice.

# B.6. Spatial impacts and delta-method inference

Because the preferred model is spatial autoregressive, the coefficient $\beta_{j}$ is not itself the marginal effect of covariate $x_{j}$. The relevant impact matrix in period $t$ is

$$R_{j,t}\left( \rho\right)=\beta_{j}\left( I_{n_{t}}-\rho W_{t} \right)^{-1}$$

For each period, the average direct effect is the mean of the diagonal elements of $R_{j,t}\left( \rho\right)$, the average total effect is the mean row sum, and the average indirect effect is the difference between total and direct effects. These quantities are then averaged across periods.

Standard errors are obtained by the delta method. Let $g(\theta)$ denote any average impact measure. Then

$$\mathrm{Var}\left( g\left( \hat{\theta} \right) \right)\approx\nabla g\left( \hat{\theta} \right)'\hat{V} \nabla g\left( \hat{\theta} \right),$$

where $\hat{V}$ is the estimated variance-covariance matrix of the SAR-IV-GMM-HAC estimator, nd

$$\nabla g\left( \hat{\theta} \right)=\left. \frac{\partial g\left( \theta\right)}{\partial\theta} \right|_{\theta=\hat{\theta}}$$

denotes the gradient vector of the impact measure with respect to the parameter vector $\theta$, evaluated at $\hat{\theta}$. The derivative of the inverse operator with respect to $\rho$ is

$$\frac{\partial}{\partial\rho}\left( I_{n_{t}}-\rho W_{t} \right)^{-1}=\left( I_{n_{t}}-\rho W_{t} \right)^{-1}W_{t}\left( I_{n_{t}}-\rho W_{t} \right)^{-1},$$

which is used to construct the gradients of the average direct, indirect, and total effects.

# B.7. Finite-sample considerations

The results in this appendix are asymptotic. In particular, the Moran and LM diagnostics, as well as the HAC-GMM estimator, are justified here through large-sample arguments. Their finite-sample performance in short unbalanced panels is not examined formally in this paper and remains an avenue for future research.

The robustness exercises reported in the main text and supplementary tables should therefore be interpreted as practical checks on sensitivity rather than as a substitute for a dedicated finite-sample study.

# References

Andrews, Donald W. K. 1991. “Heteroskedasticity and Autocorrelation Consistent Covariance Matrix Estimation.” *Econometrica* 59 (3). https://doi.org/10.2307/2938229.

Anselin, Luc, Anil K. Bera, Raymond Florax, and Mann J. Yoon. 1996. “Simple Diagnostic Tests for Spatial Dependence.” *Regional Science and Urban Economics* 26 (1). https://doi.org/10.1016/0166-0462(95)02111-6.

Kelejian, Harry H., and Ingmar R. Prucha. 1998. “A Generalized Spatial Two-Stage Least Squares Procedure for Estimating a Spatial Autoregressive Model with Autoregressive Disturbances.” *Journal of Real Estate Finance and Economics* 17 (1). https://doi.org/10.1023/A:1007707430416.

———. 1999. “A Generalized Moments Estimator for the Autoregressive Parameter in a Spatial Model.” *International Economic Review* 40 (2). https://doi.org/10.1111/1468-2354.00027.

LeSage, James, and R. Kelley Pace. 2011. “Introduction to Spatial Econometrics.” *Journal of the Royal Statistical Society Series A: Statistics in Society* 174 (2): 513–14. https://doi.org/10.1111/J.1467-985X.2010.00681_13.X.

Newey, Whitney K., and Kenneth D. West. 1987. “A Simple, Positive Semi-Definite, Heteroskedasticity and Autocorrelation Consistent Covariance Matrix.” *Econometrica* 55 (3): 703. https://doi.org/10.2307/1913610.
